# Prediction and validation of common targets in atherosclerosis and non-small cell lung cancer influenced by atorvastatin

**DOI:** 10.1186/s12906-023-04255-7

**Published:** 2023-11-17

**Authors:** Yu-qian Li, Lu-yao Li, Xue Yang, Qi-qi Lei, Liu-yan Xiang, Yuan-ru Wang, Si-meng Gu, Ya-jun Cao, Yan Pan, Lu Tie, Xue-jun Li

**Affiliations:** 1 Department of Pharmacology, School of Pharmacy, Shihezi University, Shihezi, 832002 China; 2 Department of Pharmacology, School of Basic Medical Sciences, Peking University, Beijing, 100191 China

**Keywords:** Atorvastatin, Atherosclerosis, Non-small cell lung cancer, Migration, Network pharmacology

## Abstract

**Background:**

Cardiovascular disease and cancer are the main causes of morbidity and mortality worldwide. Studies have shown that these two diseases may have some common risk factors. Atorvastatin is mainly used for the treatment of atherosclerosis in clinic. A large number of studies show that atorvastatin may produce anti-tumor activities. This study aimed to predict the common targets of atorvastatin against atherosclerosis and non-small cell lung cancer (NSCLC) based on network pharmacology.

**Methods:**

The target genes of atherosclerosis and NSCLC were obtained from The Cancer Genome Atlas (TCGA) and Gene Expression Omnibus (GEO) databases. The disease–target–component model map and the core network were obtained using Cytoscape 3.7.1. The MTS and wound healing assay were used to detect the effect of atorvastatin on cell viability and migration of A549 cells. The expression of potential common target genes of atorvastatin against atherosclerosis and NSCLC were confirmed in A549 cells and lung cancer tissues of patients.

**Results:**

We identified 15 identical pathogenic genes, and four of which (MMP9, MMP12, CD36, and FABP4) were considered as the key target genes of atorvastatin in anti-atherosclerosis and NSCLC. The MTS and wound healing assays revealed that atorvastatin decreased A549 cells migration significantly. Atorvastatin markedly decreased the expression of MMP9, MMP12, CD36, and FABP4 in A549 cells and patients were treated with atorvastatin.

**Conclusions:**

This study demonstrated 15 common pathogenic genes in both atherosclerosis and NSCLC. And verified that MMP 9, MMP 12, CD 36 and FABP 4 might be the common target genes of atorvastatin in anti-atherosclerosis and NSCLC.

**Supplementary Information:**

The online version contains supplementary material available at 10.1186/s12906-023-04255-7.

## Introduction

Recent studies have found that cardiovascular diseases and malignant tumors share several common pathogenic genes. At the same time, the two diseases often coexist in the same patient. In industrialized nations, cancer and cardiovascular disease are the main causes of death. Up to 1 of every 10 patients with ischemic heart disease has a history of cancer, whereas 1 of every 30 patients with ischemic heart disease develops a new cancer [1]. This might be because some risk factors leading to cardiovascular diseases and malignant tumors are similar. For example, the balance between cell proliferation and apoptosis plays a key role in cell development and tissue homeostasis. Breaking this balance often leads to diseases such as cancer and atherosclerotic plaques. Similarly, inflammation and dysfunction of the extracellular matrix (ECM) also cause atherosclerosis and malignant tumors [2]. At present, lung cancer is the malignant tumor with the highest mortality rate in the world. Therefore, it is worth studying the pathogenesis of atherosclerosis and cancer by exploring common pathogenic genes. Non-small cell lung cancer (NSCLC) accounts for 85% of lung cancers, and the 5-year overall survival rate is only 18% [3]. Current therapeutic methods include surgery, radiotherapy, chemotherapy, and targeted therapy. However, due to recurrence and metastasis, the overall 5-year survival rate of lung cancer patients is still relatively low. Statins have been widely used in the clinical treatment of atherosclerosis. Recent studies have shown that statins have the potential to inhibit cancer and reduce the risk of cancer [4]. Huang et al. [5] found that long-term statin therapy reduced mortality from lung cancer and dyslipidemia. Recent studies have found that statins inhibit angiogenesis and induce apoptosis in cancer cells [6]. Our previous studies showed that atorvastatin decreased the expression of vascular endothelial growth factor and inhibited angiogenesis in NSCLC by inhibiting the production of reactive oxygen species in vivo and in vitro [7]. We also found that atorvastatin can increase the sensitivity of NSCLC cells to carboplatin by inhibiting protein kinase B (Akt) activation and up-regulating Recombinant Tissue Inhibitors of Metalloproteinase 1 (TIMP1) expression, thus increasing the survival time of NSCLC tumor-bearing mice [8]. In addition, atorvastatin has been proved to inhibit the metastasis and invasion of breast cancer and prostate cancer [9]. It has been proved that atorvastatin therapy induced apoptosis in A549 cells, atorvastatin induced cell cycle arrest at G2/M phase in A549 cells. Dose-dependent treatment with atorvastatin consider ably increased caspase-3 and caspase-7 activity, indicating the induction of apoptosis [10]. Overall, these findings suggest that atorvastatin might have therapeutic potential for NSCLC. However, systematic studies on the common target genes of atorvastatin in improving atherosclerosis and NSCLC have not been reported or clarified. In this study, we mainly used a network pharmacology approach to predict potential common genes and signaling pathways of atorvastatin against atherosclerosis and NSCLC. We then used NSCLC A549 cell line and lung cancer tissue samples from atorvastatin-treated or untreated patients to validate common pathogenic genes and regulatory mechanisms. We hope to find common pathogenic genes for cardiovascular disease and NSCLC and potential targets of atorvastatin to provide clues for the treatment of both diseases.

## Methods

### Data mining of atorvastatin targeted genes

Putative targets of atorvastatin were collected from six databases using the keyword “atorvastatin,“ species were selected as “Homo sapiens” with probability > 0 as the screening condition, including the databases DrugBank [11], STITCH [12], Comparative Toxicogenomics Database [13], Similarity ensemble approach database [14], SwissTarget Prediction [15], and PharmMapper [16].

### Identification of differentially expressed genes (DEGs) in Atherosclerosis and NSCLC

The RNA-seq files of the “TCGA-LUAD” and the “TCGA-LUSC” data sets were downloaded from the TCGA database [17]. The EdgeR package was used to analyze the DEGs of NSCLC, and the cutoff value and *p* value of fold change were 1.5 and 0.05, respectively. We selected the GSE43292 data set from the GEO database [18], and GEO2R was used to identify significant DEGs based on |log_2_ (FC)| > 1 and *p* < 0.05. DEGs were mapped into a heatmap using the R heatmap package.

### Target genes of atorvastatin in treating Atherosclerosis and NSCLC

We used Cytoscape software to overlay atherosclerotic DEGs, NSCLC DEGs, and putative targets of atorvastatin to establish an interaction network so as to get coincidence genes of atorvastatin in treating atherosclerosis and NSCLC. UALCAN database was used to explore the expression of common targets in NSCLC and the expression of common targets in different cancers obtained from the Oncomine database [19] (Supplementary Material 3).

### Functional enrichment analysis

The STRING database was used to construct the protein–protein interaction networks of common targets, and it is visualized by Cytoscape 3.7.1. The Cytoscape plug-in Bisogenet was used to evaluate the topological properties of the networks [20]. Metascape [21] was used to enrich genes for KEGG pathway analysis and GO analysis [22–24].

### Survival analysis

Analysis of survival was performed using the Kaplan–Meier plotter and OncoLnc database to explore whether the aforementioned common genes were associated with prognostic significance. A threshold of *p* < 0.05 was used to set cutoff criteria.

### Cell culture and cell viability

A549 human NSCLC cell line and human liver cancer HepG2 cells were obtained from the Shanghai Institute of Biochemistry and Cell Biology (Shanghai, China). A549 cells and HepG2 cells were cultured in Dulbecco’s modified Eagle’s medium/nutrient mixture F-12 (DMEM/F12) containing 10% fetal bovine serum and 1% penicillin–streptomycin. All cells were cultured at 37 °C and 5% CO_2_. The MTS assay and CCK8 assays were used to detect cell viability. A549 cells were harvested into 96-well plates at a concentration of 5000 cells/well. After 24 h, the cells were treated with different concentrations of atorvastatin for 24 h and then incubated with 20 µL of MTS solution for 2 h. CCK8 assays were performed according to the manufacturer’s instructions. The optical density of each group was measured at 490 nm using a microplate analyzer.

### Wound healing assay

A549 cells were counted in complete medium, spread on a six-well culture plates, and cultured overnight. Vertical scratches were created using a 200 µL pipette tip. The cells in six-well plates were washed with phosphate-buffered saline (PBS), and PBS was replaced with atorvastatin-containing cell culture and control media. After 0 and 24 h, The same part of the wound was photographed under an inverted microscope, and the inhibitory effect was evaluated by ImageJ, and the data were analyzed.

### Western blot analysis

In RIPAlysis buffer containing 1% PMSF, A549 cells were lysed on ice for 20 min, and the supernatant was collected by centrifugation. Total protein concentration was measured by BCA kit, the same amount (20 µ g) of protein was separated by 12% SDS-PAGE gel and then transferred to the PVDF membrane, which was cut into different bands according to the molecular weight of the protein. The membrane was then washed with TBS-0.1% Tween buffer and blocked with 5% bovine serum albumin at room temperature for 90 min. Next, the PVDF membrane was combined with the first antibodies against MMP12 (Cat. No. 22989-1-AP), MMP9 (Cat. No. AF5228), CD36 (Cat. No. bs-1100R), FABP4 (Cat. No. DF6035), and GAPDH (Cat. No. AF7021)) at a 1:1000 dilution overnight at 4 °C. The membrane was incubated with HRP-conjugated secondary antibody (goat anti-rabbit immunoglobulin G; Cat No. ZB-2301) at a 1:10,000 dilution for 1 h at room temperature. Then the membrane was washed again, and the ECL detection kit (Cat No. KF005) was used.

### Immunohistochemistry (IHC) analysis

We collected paraffin-embedded tumor tissue samples from 21 patients with NSCLC and corresponding adjacent non-cancerous tissue samples from surgical specimens for IHC studies. This study was approved by the Ethics Committee of the First Affiliated Hospital of Shihezi University Medical College. The tissue sections were rehydrated and heat-mediated antigen repair was carried out with sodium citrate by microwave, followed by treatment with 3% hydrogen peroxide. Next, the slides were incubated with antibodies obtained in a humidified chamber at 4 °C overnight. Next, the first antibody enhancer was added drop by drop and incubated at 37 °C for 30 min. The HRP polymer was dropped, and the color reaction was achieved using DAB substrate kit for 3 min and nuclear counter staining with hematoxylin. The expression level of protein was evaluated based on staining intensity and 200× degree of staining under a microscope. The IHC intensity was measured using ImageJ 2.0 software.

### Statistical analyses

All data were expressed as mean ± standard deviation. The data were analyzed using SPSS Statistics 19.0 software (IBM Corporation, NY, USA) and GraphPad Prism V8.01 (GraphPad, CA, USA). For multiple comparisons, one-way analysis of variance was performed. The following *p* values indicated a statistically significant difference: ^*^p < 0.05; ^**^p < 0.01.

## Results

### Target genes collection of atorvastatin

We collected 5 potential atorvastatin targets from the DrugBank database, 10 potential atorvastatin targets from the STITCH database, 161 potential atorvastatin targets from the CTD database, 24 potential atorvastatin targets from the SEA database, 100 potential atorvastatin targets from the Swiss Target Prediction database. database, and 207 potential atorvastatin targets from the PharmMapper database. After eliminating redundancy, 431 potential targets of atorvastatin were collected (Table S1).

### Target genes of atorvastatin for anti-atherosclerosis and NSCLC were predicted

**Fig. 1:**
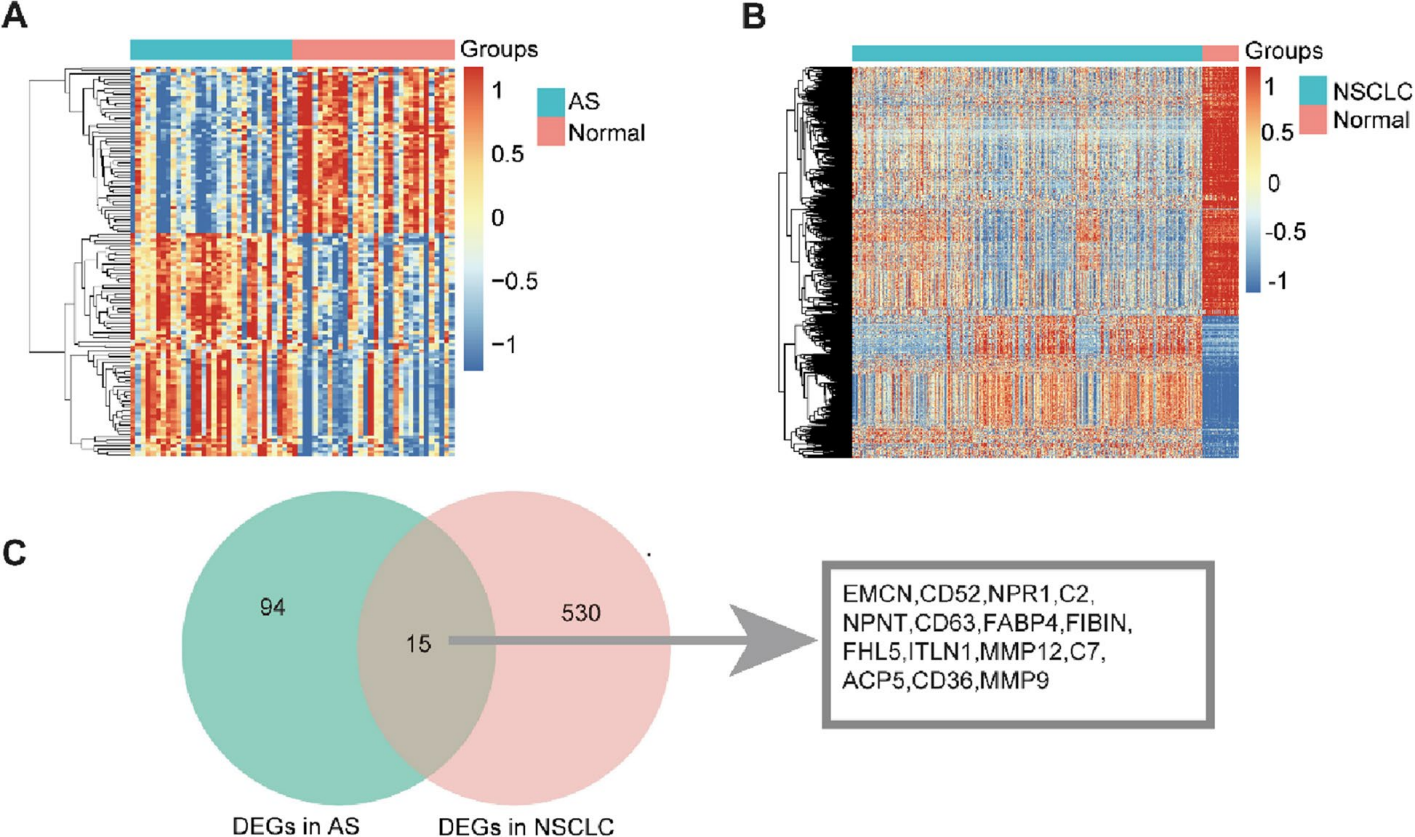
DEGs of atherosclerosis and DEGs of NSCLC, and potential common pathogenic genes of atherosclerosis and NSCLC. **A** Heat map of DEGs in atherosclerosis: red represents higher gene expression and blue represents lower gene expression. **B** Heat map of DEGs in NSCLC: red represents an over expressed gene and blue represents low gene expression. **C** Overlapping DEGs of atherosclerosis and DEGs of NSCLC. The network revealed the number of shared and unique genes of atherosclerosis and NSCLC

**Fig. 2:**
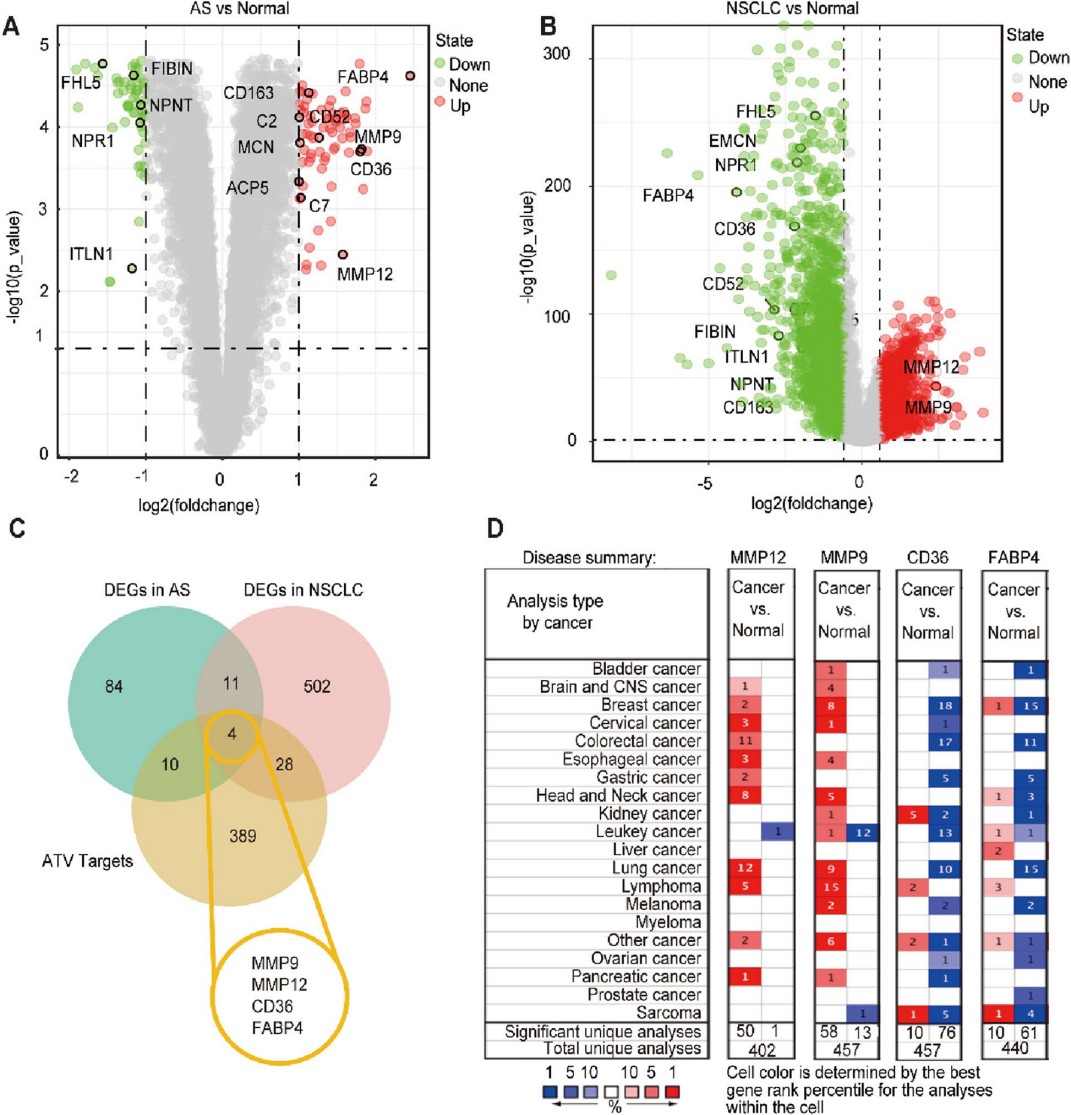
Key target genes of atorvastatin against atherosclerosis and NSCLC. **A** Volcano map of the expression of 15 identical pathogenic genes between normal tissues and atherosclerosis groups in the GEO database: the red represents the up-regulated genes in atherosclerosis tissues, and the green represents the down-regulated genes. **B** Volcano map of the expression of 15 same pathogenic genes in NSCLC; The red represents the upregulated genes in the NSCLC tissues and the green represents the down regulated genes. **C** Find 4 genes (MMP9, MMP12, CD36, and FABP4) were as potential therapeutic targets of atorvastatin for atherosclerosis and NSCLC through overlapped the genes of atorvastatin related targets, DEGs of atherosclerosis and DEGs of NSCLC. **D** Expression of four common genes associated with atorvastatin against atherosclerosis and NSCLC in various cancers

The data set GSE43292 was downloaded from the GEO database, and 109 DEGs of atherosclerosis were screened (Fig. 1A and Table S2). The RNA-seq data for “TCGA-lung adenocarcinoma (LUAD)” and “TCGA-lung squamous cell carcinoma (LUSC)” samples (*n* = 999) and 103 adjacent normal controls were downloaded from the TCGA data portal, and 545 DEGs were screened by using the R software package edge (Fig. 1B and Table S3). Fifteen overlapping DEGs of atherosclerosis and NSCLC were observed, which were the potential common pathogenic genes between atherosclerosis and NSCLC (Fig. 1C). Among these 15 pathogenic genes, MMP12, MMP9, CD36, FABP4, C7, ACP5, EMCN, CD52, C2, and CD163 were overexpressed in atherosclerosis (Fig. 2A). MMP12 and MMP9 were overexpressed in NSCLC (Fig. 2B). After overlapping the 431 putative targets of atorvastatin with 15 common pathogenic genes between atherosclerosis and NSCLC, then find 4 genes (MMP9, MMP12, CD36, and FABP4) were identified as potential therapeutic targets of atorvastatin for atherosclerosis and NSCLC (Fig. 2C). The Oncomine 4.5 database was used to explore the expression of four common targets in various cancers. The results showed that the expression of MMP9 and MMP12 in lung cancer tissues was higher than that in normal tissues adjacent to cancer, and the expression of CD36 and FABP4 in lung cancer tissues was lower than that in normal tissues adjacent to cancer (Fig. 2D).

### Functional enrichment of common genes on atorvastatin against Atherosclerosis and NSCLC

**Fig. 3:**
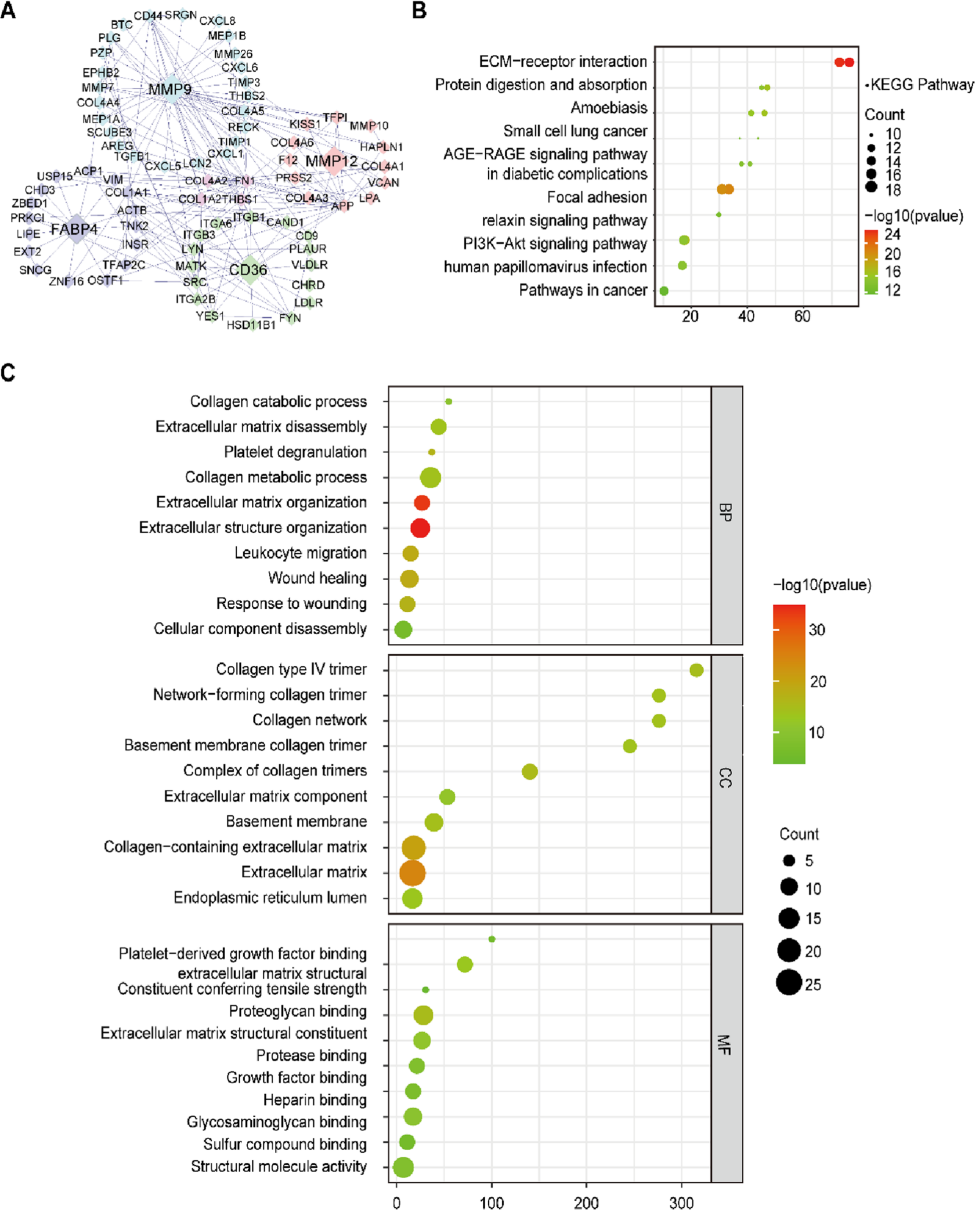
Topological properties, KEGG enrichment analysis and GO enrichment analysis. **A** The protein–protein interaction networks of MMP9, MMP12, CD36, and FABP4 of common genes were constructed by using Bisogenet in Cytoscape software. **B** KEGG analysis of top 10 enriched signaling pathways with *p* value < 0.05. **C** Top 10 signaling pathways of biological process, cell function, and molecular function in GO enrichment analysis with *p* < 0.05

Gene enrichment analysis was performed using the Metascape database to examine the function of atorvastatin against atherosclerosis and NSCLC, including Kyoto Encyclopedia of Genes and Genomes (KEGG) and Gene Ontology (GO) analyses. The protein–protein interaction networks of MMP9, MMP12, CD36, and FABP4 of common genes were constructed by using Bisogenet in Cytoscape software (Fig. 3A). KEGG pathway analysis showed that the genes were mainly correlated with ECM–receptor interaction signaling pathway, activation signaling pathway, and proteoglycans in cancer signaling pathway (Fig. 3B). GO entries were categorized into three separate groups, such as molecular function (MF), biological process (BP), and cell composition (CC). The MF classification showed that these genes were mostly enriched in the combination of extracellular structure and growth factor. The BP classification showed that these genes were mostly concentrated in the extracellular structure and wound healing. The CC category showed that the genes were mostly enriched in ECM and platelet alpha-granules (Fig. 3C).

### Expression level of common targets in NSCLC was confirmed by using the UALCAN database

**Fig. 4:**
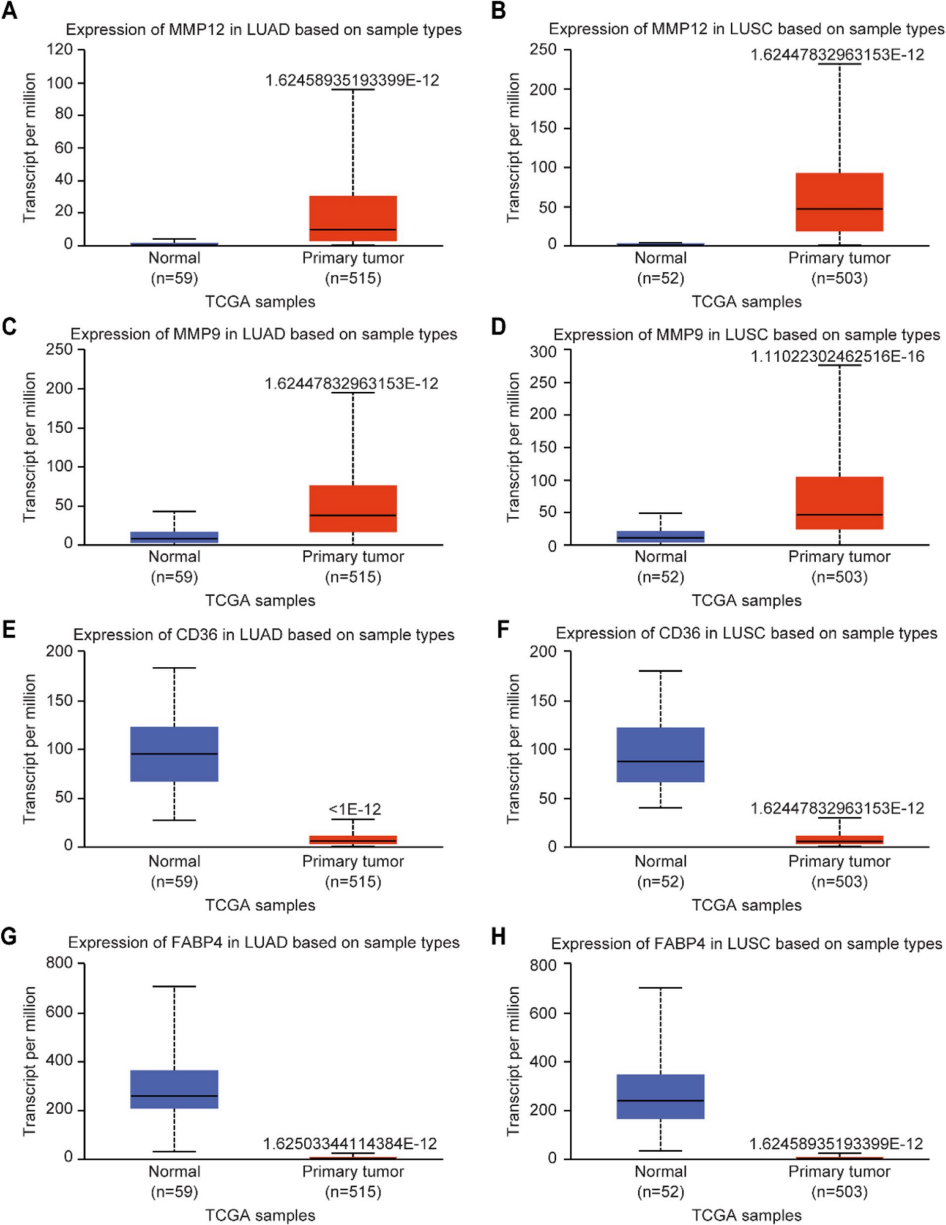
Expression of MMP12, MMP9, CD36, and FABP4 in NSCLC. **A** MMP12 was overexpressed in LUAD. **B** MMP12 was overexpressed in LUSC. **C** MMP9 was overexpressed in LUAD. **D** MMP9 was overexpressed in LUSC. **E** CD36 was underexpressed in LUAD. **F** CD36 was underexpressed in LUSC. **G** FABP4 was underexpressed in LUAD. **H** FABP4 was underexpressed in LUSC

The expression levels of four common target genes in NSCLC were verified by using the UALCAN database. The results showed that MMP12 and MMP9 were highly expressed in LUAD and LUSC tissues compared with normal tissues (Fig. 4A–D). CD36 and FABP4 were expressed at lower levels in LUAD and LUSC (Fig. 4E–H).

### Expression of MMP9, MMP12, FABP4, and CD36 was correlated with the overall survival in NSCLC using Kaplan–Meier plotter and OncoLnc database

**Fig. 5:**
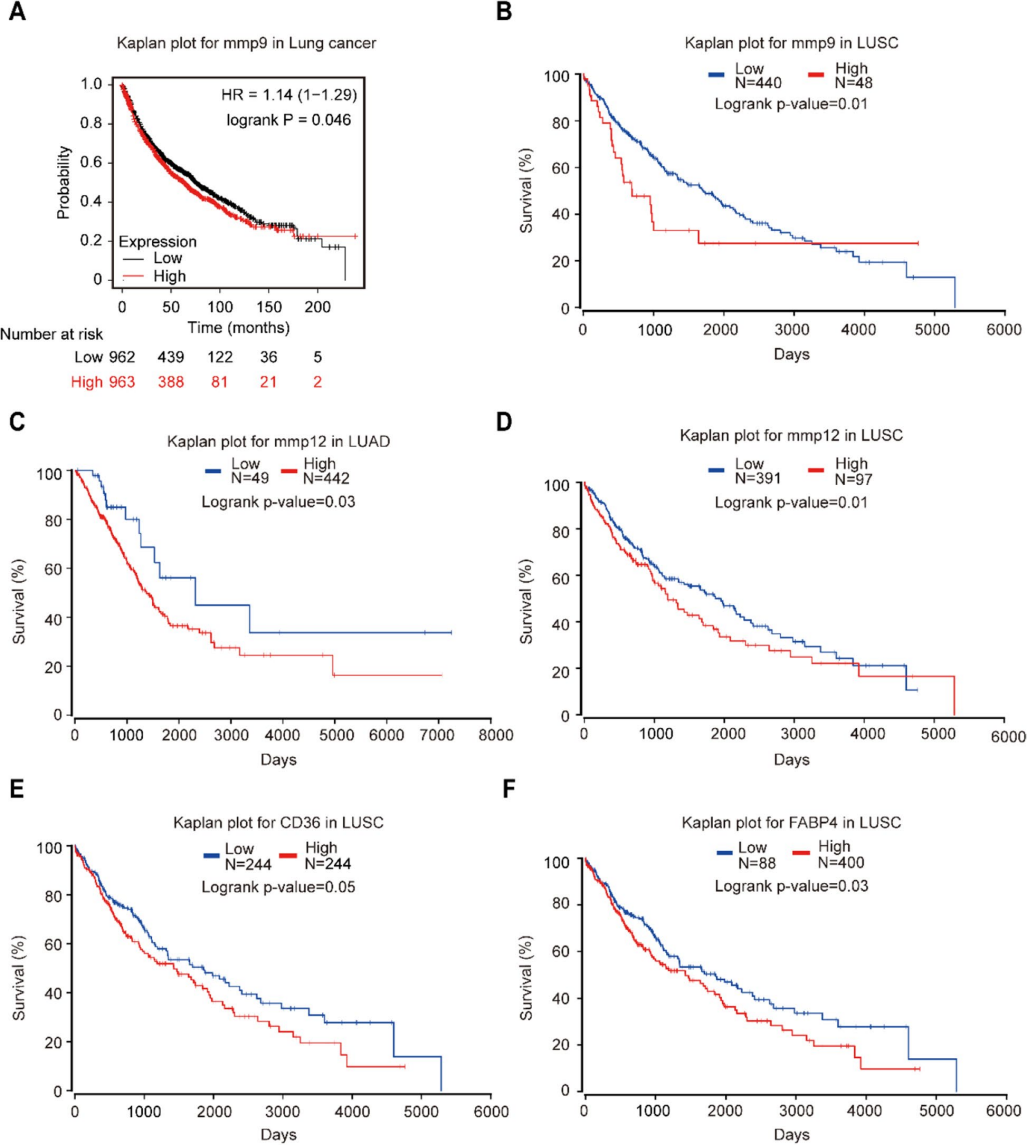
The relationship between overall survival and MMP9, MMP12, CD36, and FABP4 expression in patients with lung cancer. **A** MMP9 high expression was significantly correlated with lower overall survival (OS) rates in patients with lung cancer (*p* = 0.046). **B** The high expression of MMP9 was significantly correlated with the low incidence of OS in patients with LUSC (*p* = 0.01). **C** The high expression of MMP12 was significantly correlated with low OS rates in patients with LUAD (*p* = 0.03). **D** The high expression of MMP12 was significantly correlated with lower OS rates in patients with LUSC (*p* = 0.01). **E** The high expression of CD36 was significantly correlated with lower OS rates in patients with LUSC (*p* = 0.05). **F** The high expression of FABP4 was significantly correlated with lower OS rates in patients with LUSC (*p* = 0.03)

Kaplan–Meyer plotter and OncoLnc database were used to evaluate the correlation between the expression of MMP9, MMP12, FABP4, CD36 and NSCLC patient survival rates. The results showed that the overall survival rate was lower in patients with lung cancer and LUSC with high MMP9 expression (*p* = 0.046 and *p* = 0.01, respectively) (Fig. 5A and B). Patients with higher MMP12 expression had significantly poor overall survival in LUAD and LUSC (*p* = 0.03 and *p* = 0.01, respectively) (Fig. 5C and D). Among patients with LUSC, the overall survival rate with high expression of CD36 was obviously lower (*p* = 0.05) (Fig. 5E); and patients with LUSC having higher FABP4 expression had poor overall survival rate (*p* = 0.03) (Fig. 5F). The overexpression of MMP9, MMP12, CD36, and FABP4 may serve as a novel indicator of reduced survival time in patients with NSCLC. Therefore, MMP9, MMP12, CD36, and FABP4 may be used as prognostic biomarkers for LUSC.

### Inhibitory effects of atorvastatin on the cell viability of A549 cells

**Fig. 6:**
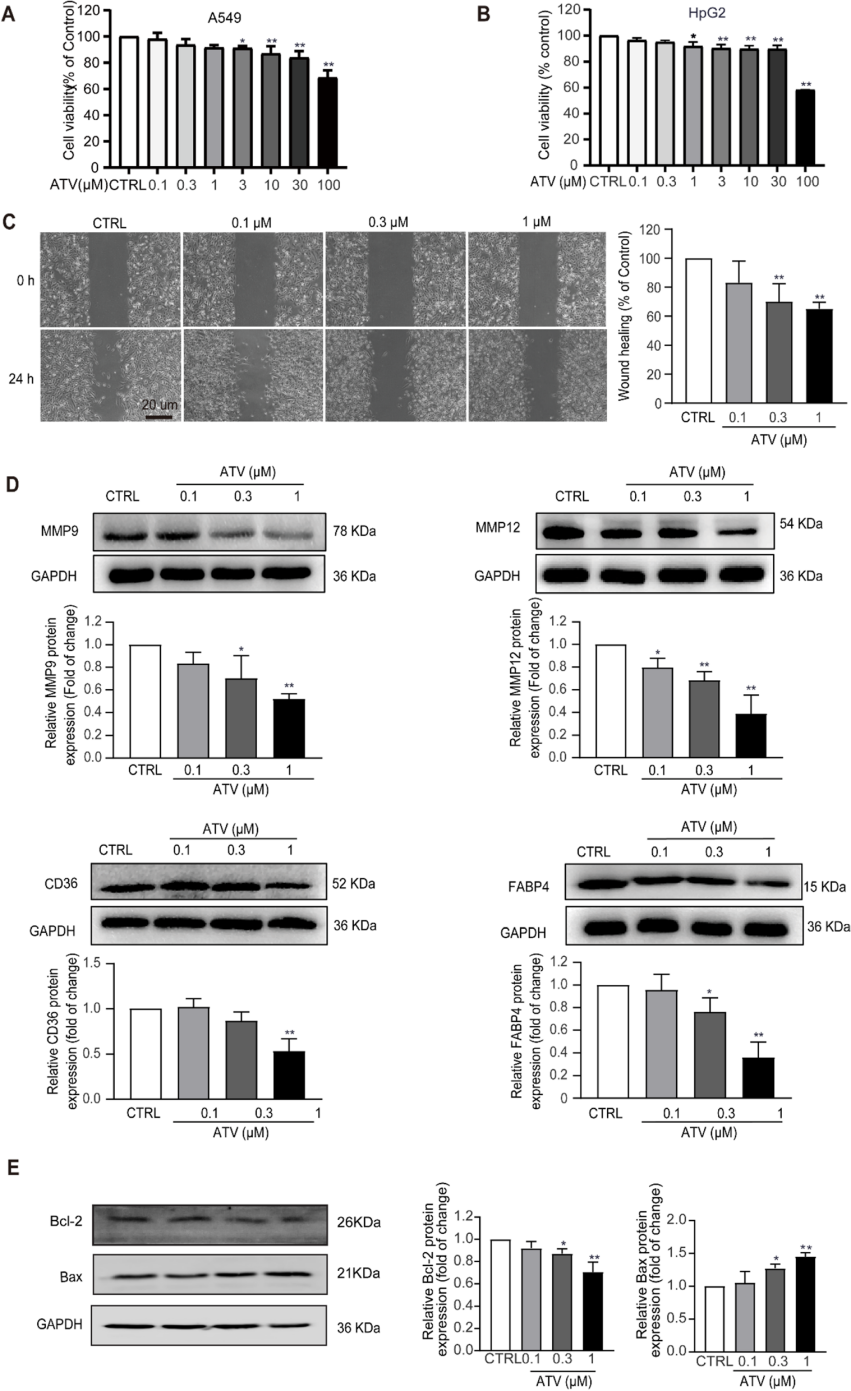
Effects of atorvastatin on the proliferation and migration of A549 cells. **A** A549 cells were exposed to atorvastatin at concentrations of 0, 0.1, 0.3, 1, 3, 10, 30, and 100 µM for 24 h, and then MTS assays was performed to determine the viability of the cell. Atorvastatin inhibited the proliferation of A549 cells in a dose-dependent manner. **B**  HepG2 cells were exposed to atorvastatin at concentrations of 0, 0.1, 0.3, 1, 3, 10, 30, and 100 µM for 24 h, and then CCK8 assays was performed to determine the viability of the cell. Atorvastatin inhibited the proliferation of HepG2 cells in a dose-dependent manner. **C** A549 cells were treated with atorvastatin at concentrations of 0, 0.1, 0.3, and 1 µM for 24 h. Results of the wound healing assay showed that cell healing over scratch was inhibited by the treatment of atorvastatin. The result indicated that atorvastatin therapy reduced the migration of A549 cells in a dose-dependent manner. Scale bar = 50 μm. **D** Effects of atorvastatin on the activities of MMP9, MMP12, CD36, and FABP4 in A549 cells. Western blot was used to evaluate the expression of MMP9, MMP12, CD36, and FABP4. The result indicated that atorvastatin significantly reduced the expression of MMP9, MMP12, CD36, and FABP4 in a concentration-dependent manner. **E** Atorvastatin treatment significantly upregulated the expression of pro-apoptosis protein Bax and downregulated the anti-apoptosis protein Bcl-2 protein. * *p*  < 0.05, ** *p*  < 0.01 vs. control, *n*  = 3. Scale bars = 20 μm

The MTS analysis was performed to evaluate the effect of atorvastatin on the viability of A549 cells, and the optimal concentration of atorvastatin was selected for further study. A549 cells were exposed to different concentrations of atorvastatin (0, 0.1, 0.3, 1, 3, 10, 30, and 100 µM) for 24 h. Atorvastatin at concentrations of 10, 30, and 100 µM significantly decreased the *viability* of A549 cells (*p* < 0.05) (Fig. 6A). At the same time, atorvastatin can also inhibit the proliferation of human liver cancer HepG2 cells in a concentration-dependent manner (Fig. 6B).

### Effects of atorvastatin on the migration of A549 cells detected using wound healing assay

We used a scratch wound healing test to further examine the potential of atorvastatin in inhibiting the migration of A549 cells. A549 cells were seeded in six-well plates, and wounding on the next day was exposed to atorvastatin at 0, 0.1, 0.3, and 1 µM. Cell motility was monitored under a microscope at 0 and 24 h after the wound was produced. After 24 h, compared with the control group, the doses of 0.3 and 1 µM significantly inhibited migration (Fig. 6C). These results showed that atorvastatin could significantly inhibit the migration of A549 cell.

### Effects of atorvastatin on the protein expression detected using Western blot assay

We further studied the effect of atorvastatin on the expression of MMP9, MMP12, CD36, and FABP4 in A549 cells in order to explore the possible underlying mechanism of atorvastatin inhibiting the migration of NSCLC. In the present study, different concentrations of atorvastatin (0.1, 0.3, and 1 µM) significantly decreased the protein expression of MMP9, MMP12, CD36, and FABP4 in A549 cells (*P* < 0.05). Atorvastatin at doses of 0.3 and 1 µM significantly decreased the expression of CD36 (Fig. 6D). These results suggested that the reduction in protein expression of MMP12, MMP9, CD36, and FABP4 played a key role in the inhibitory effect of atorvastatin on NSCLC cell migration. To verify whether atorvastatin can promote the apoptosis of A549 cells. We detected the expression of apoptosis-related proteins: anti-apoptosis protein Bcl-2 and increasing apoptosis protein Bax. Our results showed that atorvastatin significantly up-regulated the expression of pro-apoptotic protein Bax and down-regulated the expression of anti-apoptotic protein Bcl-2 (Fig. 6E).

### Evaluation of the expression of MMP9, MMP12, CD36, and FABP4 in patients with LUAD and LUSC with or without atorvastatin administration

**Fig. 7:**
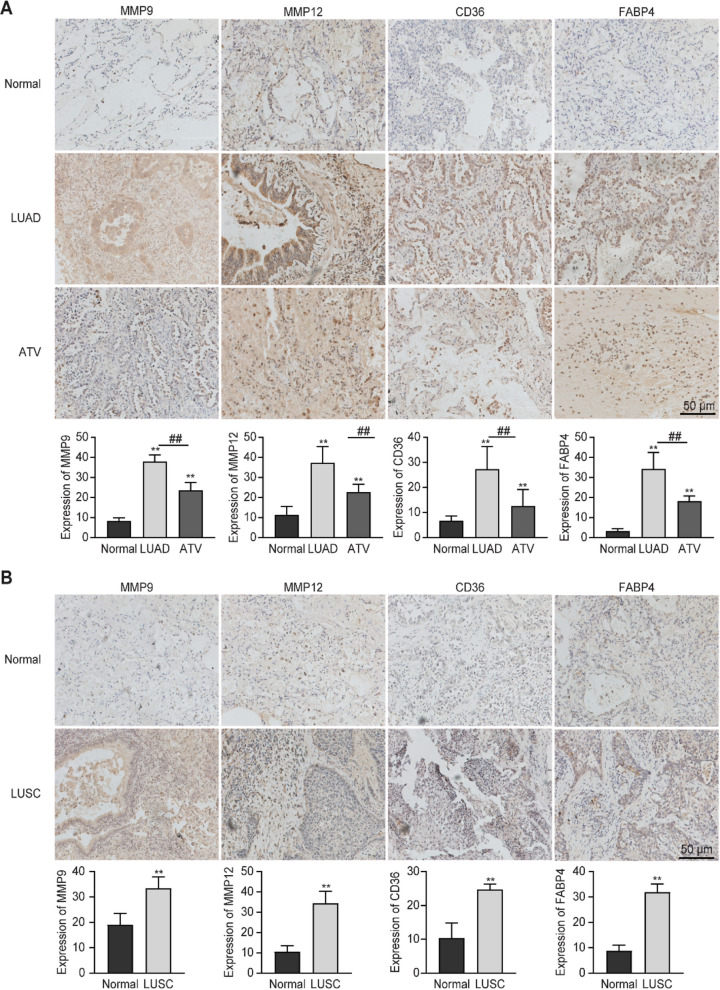
Immunohistochemistry staining determined MMP12, MMP9, CD36, and FABP4 expression in tissues of patients with NSCLC. **A** Compared with the adjacent non-cancerous lung tissues of patients taking atorvastatin, MMP12 and MMP9 were highly expressed in the tumor stroma of LUAD. CD36 presented a cell membrane pattern of staining, and the expression levels of CD36 in LUAD were higher compared with adjacent non-cancerous lung tissues in patients who took atorvastatin. FABP4 showed nuclear and cytoplasmic staining patterns, and the expression levels of CD36 in LUAD of patients taking atorvastatin was higher compared with adjacent noncancerous lung tissues. **B** Expression of MMP12, MMP9, CD36, and FABP4 expression in LUSC tissue samples and corresponding adjacent non-cancerous lung tissues. In patients taking atorvastatin, MMP12, MMP9, CD36, and FABP4 were highly expressed in the LUSC, butwere low expressed in the adjacent non-cancerous lung tissues. The intensity of immunohistochemical staining was analyzed using ImageJ 2.0 software. ^*^p < 0.05, ^**^p < 0.01 vs. control. ^##^*p* < 0.01 vs. ATV. Scale bars = 50 μm

We detected the expression of MMP9, MMP12, CD36, and FABP4 in the tissues of patients. In all cases, IHC staining showed a cytoplasmic staining pattern for MMP12 and MMP9, FABP4 showed a nuclear and cytoplasmic staining, and CD36 showed a membrane staining pattern. Representative IHC images showed higher expression of MMP12 and MMP9 in the tumor stroma of LUAD compared with adjacent noncancerous lung tissues and tissues from patients taking atorvastatin (Fig. 7A). Compared with the adjacent normal tissues and cancer tissues of patients taking atorvastatin, the expressions of MMP9, MMP12, CD36, and FABP4 in LUSC tissues increased significantly (Fig. 7B).

## Discussion

Atherosclerosis and cancer have many similar pathogenesis, and many common risk factors have been found in the occurrence and development of these two diseases [25]. Atherosclerosis is characterized by intimal thickening and plaque formation of arterial [26]. Concurrent inflammation and ECM dysfunction are common pathogenesis of these two diseases. As lipid-lowering drugs, statins are widely used to reduce cholesterol levels to prevent cardiovascular events, but the relationship between statins and cancer is still controversial. Previous studies have shown that atorvastatin could inhibit the proliferation of breast cancer, pancreatic cancer, and prostate cancer cells in vitro by inducing apoptosis and autophagy [27]. Our previous research found that atorvastatin synergized with IFN-γ to inhibit the growth of NSCLC cells by reducing Rho activity [28]. Therefore, atorvastatin deserves further study in terms of potential adjuvant therapy for malignant tumors.

Based on the method of network pharmacology, we found that there were 15 common genes in atherosclerosis and NSCLC, including CD163, MMP9, ACP5, FABP4, EMCN, CD52, NPR1, MMP12, C2, ITLN1, FHL5, CD36, FIBIN, C7, and NPNT. Among them, MMP12, C7, ACP5, CD36, EMCN, MMP9, CD52, C2, CD163, and FABP4 were highly expressed in atherosclerosis. MMP12 and MMP9 were highly expressed in NSCLC. After the potential target genes of atorvastatin interact with more than 15 common genes in the aforementioned two diseases, we obtained four genes: MMP9, MMP12, CD36, and FABP4. Further analysis using the UALCAN database showed that MMP9, MMP12, CD36, and FABP4 were over expressed in atherosclerotic tissues, while the expression of MMP9 and MMP12 were up-regulated in NSCLC tissues and the expression of CD36 and FABP4 were down-regulated in NSCLC tissues. The pathway analysis showed that these genes were involved in ECM–receptor interaction pathway, platelet activation pathway, and proteoglycan in tumor pathway. Therefore, the therapeutic effect of atorvastatin on atherosclerosis and NSCLC may be on the ECM, which needs validation via further studies.

Fatty acid binding protein 4 (FABP4) is a member of the intracellular lipid binding protein family, which is mainly expressed in adipocytes, macrophages, and endothelial cells. As a fatty acid transporter, FABP4 plays an important role in the uptake, transport, and metabolism of long-chain fatty acids and is closely related to atherosclerosis [29]. In this study, FABP4 was highly expressed in atherosclerosis, which was consistent with existing reports. Studies showed that FABP4 was associated with a high-risk phenotype of atherosclerotic plaques, including inflammatory and vulnerable plaques [30]. Llaverias et al. [31] showed that atorvastatin alleviated atherosclerosis by inhibiting the expression of FABP4. Therefore, reducing FABP4 might be a potential treatment for atherosclerosis. The tumor microenvironment plays a crucial role in tumor growth and metastasis. Adipocytes provide fatty acids to meet the high energy requirement of tumor cell proliferation and invasion. This study showed that the expression of FABP4 was low in the tissues of patients with NSCLC. There was a significant difference in the expression of FABP4 between primary and metastatic tumors. Typically, FABP4 expression was higher in metastatic tumors but lower in primary tumors. This was probably due to FABP4, a mediator of lipid transport in adipocytes and tumor-initiating cells. Adipocytes provide fatty acids to cancer cells, thus promoting rapid growth and metastasis of tumor [32]. Tang et al. showed that increased FABP4 expression was closely related to advanced tumor metastasis [33]. Therefore, we hypothesized that atorvastatin inhibited the metastasis of NSCLC by downregulating the expression of FABP4. In this study, we confirmed the hypothesis and found that atorvastatin down-regulated the expression of FABP4 in A549 cells.

CD36 is a member of the scavenger receptor family, which is expressed in various tissues and cells, promoting monocyte aggregation and infiltration to inflammatory sites. CD36 is correlated with the occurrence and development of atherosclerosis [34]. In this study, we found that CD36 was highly expressed in atherosclerosis. This suggested that the inhibition of CD36 might reduce the number of macrophage foam cells. Furthermore, we found that CD36 expression was down regulated in patients with NSCLC tissues compared with adjacent normal tissues. These results are consistent with previous findings. CD36 is expressed on tumor cells, stromal cells, and immune cells, but at different levels in different cell types and tumor stages. CD36 is overexpressed in metastatic tumors, whereas the expression of CD36 is basically negative in primary tumors. Therefore, CD36 played an important role in lipid homeostasis and tumor metastasis [35]. It is generally believed that CD36 is a potential biomarker and therapeutic target for cancer. These results indicate that atorvastatin could inhibit the progression of NSCLC by inhibiting metastasis. Atorvastatin can be used as an effective adjuvant drug targeting CD36 and preventing tumor metastasis.

This study found that MMP9 is highly expressed in atherosclerosis. It was mainly released by neutrophils and macrophages, and it could degrade ECM proteins, lipoproteins and cell adhesion molecules. MMP9 could also degrade type IV collagen in the basement membrane and accelerate the rupture of plaque. As one of the important causes of plaque instability, the expression of MMP9 in unstable plaques is significantly higher than that in stable plaques [36]. Jia et al. [37] showed that the reduction in MMP9 expression by atorvastatin contributed to plaque stability. The findings of this study suggested that MMP9 was highly expressed in NSCLC tumor tissues. MMP9 plays an irreplaceable role in tumor initiation and progression, including tumor angiogenesis, promotion of tumor growth, and destruction of the basement membrane to promote tumor metastasis and spread [38]. Therefore, MMP9 can be used as a biomarker to predict the survival and prognosis of patients with NSCLC. Atorvastatin plays an important role in inhibiting the overexpression of MMP9. Studies are needed to further explore the mechanism of action of atorvastatin on MMP9 to effectively treat both atherosclerosis and NSCLC.

This study found by network analysis that MMP12 was highly expressed in both atherosclerosis and NSCLC. MMP12 was initially found in the alveolar macrophages of smokers and was mainly secreted and expressed by activated macrophages. Studies showed that the overexpression of MMP12 was associated with atherosclerosis [39]. Also, Hofmann et al. [40] showed that MMP12 was highly expressed in NSCLC tumor tissues, and the high expression of MMP12 was correlated with metastasis and high recurrence rate in patients with NSCLC. Therefore, atorvastatin might inhibit atherosclerosis and NSCLC by inhibiting MMP12. It is reported that atorvastatin can improve survival rates of various cancers. Therefore, we studied whether MMP9, MMP12, FABP4, and CD36 genes are correlated with poor prognosis in various cancers. These results suggest that the inhibition of these genes with atorvastatin might prolong survival in patients with cancer.

In conclusion, MMP9, MMP12, FABP4, and CD36 genes are closely related to the occurrence and development of atherosclerosis and NSCLC. We hypothesized that atorvastatin could inhibit the expression of MMP9, MMP12, FABP4, and CD36 in patients with atherosclerosis and NSCLC. Atorvastatin might reduce the degradation of matrix components and inflammatory reaction in atherosclerotic plaques by regulating four genes, stabilize atherosclerotic plaques, inhibit tumor metastasis and prolong patient’s survival. However, this study also had limitations. We used limited samples, leading to unexpected errors. Hence, in vivo studies are needed to validate the findings of this study.

## Conclusions

Based on network pharmacology analysis, further cell experiments in vitro and IHC analyses of samples from patients with NSCLC in this study showed that atherosclerosis and NSCLC shared some of the same pathogenic genes. The underlying molecular mechanism was mainly correlated with the regulation of the ECM–receptor interaction signaling pathway. Atorvastatin inhibits atherosclerosis and NSCLC by down-regulating the expression of MMP9, MMP12, FABP4, and CD36. MMP9, MMP12, FABP4, and CD36 might be the potential therapeutic targets in treating atherosclerosis and NSCLC.

### Supplementary Information

**Additional file 1: Supplementary Table 1.** Target genes of atorvastatin. **Supplementary Table 2.** Differentially expressed genes (DEGs) of atherosclerosis. **Supplementary Table 3.** Differentially expressed genes (DEGs) of Non small cell lung cancer(NSCLC).

** Additional file 2.** 

** Additional file 3.**

## Data Availability

The datasets used and analyzed during the current study are available from the corresponding author on reasonable request. And most data generated or analyzed during this study are included in this published article and its supplementary information files. Publicly available data are from the DrugBank (https://www.drugbank.com/) database; STITCH (http://stitch.embl.de/), Comparative Toxicogenomics Database (https://ctdbase.org/), Similarity ensemble approach database (https://sea.bkslab.org/), SwissTarget Prediction (http://www.swisstargetprediction.ch/), and PharmMapper (http://lilab-ecust.cn/pharmmapper/submitfile.html). TCGA (https://cancergenome.nih.gov/abouttcga/overview 1) database, data set from the GEO (https://www.ncbi.nlm.nih.gov/geo/) database with the accession ID GSE43292. The STRING (https://cn.string-db.org/) database. Oncomine (https://www.oncomine.org/resource/main.html) database (Supplementary Material 3). Kaplan–Meier plotter (https://kmplot.com/analysis/index.php?p=service&cancer=lung) and OncoLnc (http://www.oncolnc.org/) database.

## References

[1] Raposeiras Roubín RS, Cordero A (2019). The two-way relationship between Cancer and Atherosclerosis. Revista Esp De cardiologia (English ed).

[2] Meijers WC, De B (2019). Common risk factors for Heart Failure and cancer. Cardiovascular Res.

[3] Bray F, Ferlay J, Soerjomataram I (2018). Global cancer statistics 2018: GLOBOCAN estimates of incidence and mortality worldwide for 36 cancers in 185 countries. Cancer J Clin.

[4] Matusewicz L, Czogalla A, Sikorski AF (2020). Attempts to use statins in cancer therapy: an update. Tumor Biology.

[5] Huang WY, Li CH, Lin CL (2016). Long-term statin use in patients with Lung cancer and dyslipidemia reduces the risk of death. Oncotarget.

[6] He Z, Yuan J, Shen F (2020). Atorvastatin enhances effects of Radiotherapy on Prostate Cancer cells and xenograft Tumor mice through triggering Interaction between Bcl-2 and MSH2. Med Sci Monit.

[7] Chen J, Liu B, Yuan J (2012). Atorvastatin reduces vascular endothelial growth factor (VEGF) expression in human non-small cell lung carcinomas (NSCLCs) via inhibition of reactive oxygen species (ROS) production. Mol Oncol.

[8] Chen J, Lan T, Hou J (2012). Atorvastatin sensitizes human non-small cell lung carcinomas to carboplatin via suppression of AKT activation and upregulation of TIMP-1. Int J Biochem Cell Biol.

[9] Ma Q, Gao Y, Xu P (2019). Atorvastatin inhibits Breast Cancer cells by downregulating PTEN/AKT pathway via promoting ras Homolog Family Member B (RhoB). Biomed Res Int.

[10] Du X, Li D, Wang G (2021). Chemoprotective effect of atorvastatin against benzo(a)pyrene-induced Lung cancer via the inhibition of oxidative stress and inflammatory parameters. Annals of Translational Medicine.

[11] Wishart D, Feunang Y, Guo A (2018). DrugBank 5.0: a major update to the DrugBank database for 2018. Nucleic Acids Res.

[12] Kuhn M, von Mering C, Campillos M (2008). STITCH: interaction networks of chemicals and proteins. Nucleic Acids Res.

[13] Davis AP, Murphy CG, Saraceni-Richards CA (2008). Comp Toxicogenomics Database. Nucleic Acids Res.

[14] Keiser MJ, Roth BL, Armbruster BN (2007). Relating protein pharmacology by ligand chemistry. Nat Biotechnol.

[15] Daina A, Michielin O, Zoete V (2019). SwissTargetPrediction: updated data and new features for efficient prediction of protein targets of small molecules. Nucleic Acids Res.

[16] Xia W, Yihang S, Shiwei W (2017). PharmMapper 2017 update: a web server for potential drug target identification with a comprehensive target pharmacophore database. Nucleic Acids Res.

[17] Haussler D (2013). The Cancer Genome Atlas. Science.

[18] Ayari H, Bricca G (2013). Identification of two genes potentially associated in iron-heme homeostasis in human carotid plaque using microarray analysis. J Bioences.

[19] Chandrashekar DS, Bashel B, Balasubramanya SAH (2017). UALCAN: a portal for facilitating Tumor Subgroup Gene expression and survival analyses. Neoplasia.

[20] Franceschini A, Szklarczyk D, Frankild S (2013). STRING v9.1: protein-protein interaction networks, with increased coverage and integration. Nucleic Acids Res.

[21] Zhou Y, Zhou B, Pache L (2019). Metascape provides a biologist-oriented resource for the analysis of systems-level datasets. Nat Commun.

[22] Kanehisa M, Goto S (2000). KEGG: kyoto encyclopedia of genes and genomes. Nucleic Acids Res.

[23] Kanehisa M (2019). Toward understanding the origin and evolution of cellular organisms. Protein Science: A Publication of the Protein Society.

[24] Kanehisa M, Furumichi M, Sato Y (2023). KEGG for taxonomy-based analysis of pathways and genomes. Nucleic Acids Res.

[25] Tapia-Vieyra J, Delgado-Coello B, Mas-Oliva J (2017). Atherosclerosis and Cancer; a resemblance with Far-reaching implications. Arch Med Res.

[26] Minelli S, Minelli P, Montinari MR (2020). Reflections on Atherosclerosis: lesson from the past and future research directions. J Multidiscip Healthc.

[27] Jones HM, Fang Z, Sun W (2017). Atorvastatin exhibits anti-tumorigenic and anti-metastatic effects in Ovarian cancer in vitro. Am J Cancer Res.

[28] Chen J, Hou J, Zhang J (2012). Atorvastatin synergizes with IFN-γ in treating human non-small cell lung carcinomas via potent inhibition of RhoA activity. Eur J Pharmacol.

[29] Furuhashi M, Fuseya T, Murata M, et al. Local production of fatty acid-binding protein 4 in Epicardial/Perivascular Fat and macrophages is linked to coronary Atherosclerosis. Arterioscler Thromb Vasc Biol. 2016. ATVBAHA.116.307225. 10.1161/ATVBAHA.116.307225 27013610

[30] Bo M, Kemmerer M, Brüne B (2015). FABP4 inhibition suppresses PPARγ activity and VLDL-induced foam cell formation in IL-4-polarized human macrophages. Atherosclerosis.

[31] Llaverias G, Noé V, Peñuelas S (2004). Atorvastatin reduces CD68, FABP4, and HBP expression in oxLDL-treated human macrophages. Biochem Biophys Res Commun.

[32] Nieman KM, Kenny HA, Penicka CV (2011). Adipocytes promote Ovarian cancer Metastasis and provide energy for rapid Tumor growth. Nat Med.

[33] Tang Z, Shen Q, Xie H (2016). Elevated expression of FABP3 and FABP4 cooperatively correlates with poor prognosis in non-small cell lung cancer (NSCLC). Oncotarget.

[34] Tian K, Xu Y, Sahebkar A (2020). CD36 in Atherosclerosis: pathophysiological mechanisms and therapeutic implications. Curr Atheroscler Rep.

[35] Wang J, Li Y (2019). CD36 tango in cancer: signaling pathways and functions. Theranostics.

[36] Ye S (2006). Influence of matrix metalloproteinase genotype on Cardiovascular Disease susceptibility and outcome. Cardiovascular Res.

[37] Jia F, Wu C, Chen Z (2016). Atorvastatin attenuates atherosclerotic plaque destabilization by inhibiting endoplasmic reticulum stress in hyperhomocysteinemic mice. Mol Med Rep.

[38] Huang H (2018). Matrix Metalloproteinase-9 (MMP-9) as a Cancer Biomarker and MMP-9 biosensors: recent advances. Sens (Basel).

[39] Liang J, Liu E, Yu Y (2006). Macrophage metalloelastase accelerates the progression of Atherosclerosis in transgenic rabbits. Circulation.

[40] Hofmann HS, Hansen G, Richter G (2005). Matrix Metalloproteinase-12 expression correlates with local recurrence and metastatic Disease in non–small cell Lung Cancer patients. Clin Cancer Res Official J Am Association Cancer Res.

