# Supplementary material for: Prediction and validation of common targets in atherosclerosis and non-small cell lung cancer influenced by atorvastatin
Source: BMC Complement Med Ther. 2023 Nov 17;23:415. doi: 10.1186/s12906-023-04255-7 (PMC10657002; doi:10.1186/s12906-023-04255-7)
Supplement: Supplementary file 1 — Additional file 1: Supplementary Table 1. Target genes of atorvastatin. Supplementary Table 2. Differentially expressed genes (DEGs) of atherosclerosis. Supplementary Table 3. Differentially expressed genes (DEGs) of Non small cell lung cancer(NSCLC). [file 12906_2023_4255_MOESM1_ESM.docx]

Supplementary Materials for

**Prediction and validation of common targets in atherosclerosis and non-small cell lung cancer influenced by atorvastatin**

**Yuqian Li^1^, Luyao Li^1^, Xue Yang^1^, Qiqi Lei^1^, Liuyan Xiang^1^, Yuanru Wang^1^, Simeng Gu^2^, Yajun Cao^1^, Yan Pan^2^, Lu Tie^2^,** **Xuejun Li^1, 2*^**

^1^Department of Pharmacology, School of Pharmacy, Shihezi University, Shihezi 832002, China

^2^Department of Pharmacology, School of Basic Medical Sciences, Peking University, Beijing 100191, China

*Correspondence: Xuejun Li, Email: xjli@bjmu.edu.cn.

**Supplementary Material**

**Supplementary Table S1.**

Supplementary Table 1 Target genes of atorvastatin

| Target genes of atorvastatin | | | | | | |
| --- | --- | --- | --- | --- | --- | --- |
| ABCA1 | CDKN1B | IL2RA | NR1I3 | SLC4A1 | EGLN1 | SERPINE1 |
| ABCB1 | CHUK | IL6 | PARP1 | SLC6A4 | MAPK8 | VCP |
| ABCB11 | CLCN1 | INS | PCYT1A | SLCO1A4 | CMA1 | FKBP1A |
| ABCB4 | CRP | INS1 | PDK4 | SLCO1B1 | DDR2 | SLC6A1 |
| ABCC2 | CSF2 | ITGA2 | PDPK1 | SLCO1B3 | VDR | CASP6 |
| ABCC3 | CXCL8 | ITGAM | PLAU | SLCO2B1 | PTGFR | PLA2G2A |
| ACE2 | CYCS | ITGAX | POMC | SOD1 | GIPR | FKBP5 |
| ADAM12 | CYP27A1 | ITGB1 | PON1 | SOD2 | AKR1C3 | CFD |
| ADAM17 | CYP2B10 | JUN | PON2 | SOD3 | GRB2 | CCR1 |
| ADIPOQ | CYP3A11 | KCNH2 | PPARA | SREBF1 | PTGER1 | FKBP4 |
| AGT | CYP3A233A1 | KHK | PPARGC1A | SREBF2 | MMP1 | SRD5A2 |
| AKT1 | CYP3A4 | LCAT | PPAR | SYP | LTB4R | PIK3CB |
| APOB | DCX | LCN2 | PRKACA | TFAM | PTGIS | CCKBR |
| APOE | DGAT1 | LDLR | PRKCA | TFRC | NR1H4 | FOLR1 |
| APP | EDN1 | LEP | PRKCQ | TLR2 | PPARD | ITGAV |
| BAK1 | EGFR | LPL | PRKCZ | TLR4 | CASP1 | MMP13 |
| BAX | EIF2S1 | MAPK1 | PTEN | TNF | RARG | MMP12 |
| BCL2 | EPB41 | MAPK14 | PTGS2 | RXRA | RARB | GCG |
| BECN1 | F2 | MAPK3 | RAC1 | TNFSF10 | CPT1A | STAT5B |
| BID | F5 | MB | RB1 | TOMM20 | ITGAL | BACE2 |
| BIRC5 | FADD | MCL1 | RELA | TP53 | PDE4A | BACE1 |
| BMP2 | FAS | MDM2 | RHOA | TUBB3 | PDE4B | PTPN7 |
| C3 | FASLG | MFN2 | RHOB | VCAM1 | PDE4D | NR1H3 |
| CASP3 | FASN | MLXIPL | RHOC | VWF | LPAR3 | PTPN6 |
| CASP8 | GCK | MMP2 | SCARB1 | DPP4 | LPAR1 | PTPRC |
| CD86 | SLC22A3 | PRGR | WASP | PH4H | IL2 | MP2K1 |
| IL1B | FDFT1 | CATD | CCNA2 | DHSO | FABP6 | CATG |
| NR1I2 | EDNRB | MK01 | KC1G2 | GSK3B | CP2C9 | GSTT2 |
| SLC2A4 | LPAR2 | CAH12 | PYRD | PK3CG | PTGD2 | AK1C2 |
| ESR2 | CAH1 | DDX6 | PTN11 | PAK7 | FABP7 | DPEP1 |
| ITGA4 | CD40LG | GSTP1 | B3GA1 | PPAP | PYR5 | TPIS |
| DUSP3 | ICAM1 | NQO1 | STK6 | ERG7 | TGM3 | ARGI2 |

| Target genes of atorvastatin | | | | | | |
| --- | --- | --- | --- | --- | --- | --- |
| UROK | NOX1 | CFAB | CACP | P15086 | RB11A | MMP7 |
| CDKN1A | SLC22A5 | PPARG | DHI1 | ZA2G | S10A9 | ACADM |
| DGAT1 | NR3C1 | CD5R1 | MCR | CCNT1 | CSK | RAB5A |
| PDE5A | HRH1 | TGFR1 | EST1 | ALDH2 | SPYA | FPPS |
| PTGIR | P2RY12 | PNPH | MIF | FA7 | ITAL | FGF1 |
| PTGER3 | TTHY | CATL2 | FGFR1 | KPCT | ELNE | EPHA2 |
| CPT2 | CD83 | FABP4 | CATB | IGF1R | LGUL | KTHY |
| SLC22A6 | IKBKB | RORA | FABP5 | JAK3 | LYAM3 | SETD7 |
| CHK1 | NPPB | ANXA5 | IMPA1 | SAHH | C1S | CLK1 |
| CD14 | SLC2A2 | BRAF1 | LCK | CALM | TIE2 | TPH1 |
| HBEGF | PTGER4 | HS90A | TYSY | HXK1 | G6PI | KIT |
| NFE2L2 | PTPN1 | ANGI | ADHX | PDK2 | HXK4 | ARSA |
| SLC1A3 | EPHX2 | SRC | VGFR2 | ACK1 | GCR | TGFB2 |
| HDAC1 | AOFB | CATS | KSYK | TYPH | AMYP | TRYB2 |
| PTPN22 | CASP9 | SHBG | BAG1 | GLCM | CCL5 | IMDH1 |
| THRA | GNG3 | VTDB | MMP8 | FABPH | PLGF | RXRB |
| FA10 | MMP9 | Q16539 | LEG7 | CTNA1 | PAK6 | CCL2 |
| CD36 | SELE | DYR | HMDH | ISG20 | JAK2 | GPT |
| HMGCR | AHR | DDX6 | AK1C3 | BST1 | LEG2 | MYH2 |
| NOS2 | TYRO3 | KAT1 | PDE3B | ADA17 | ALDOA | SLC1A2 |
| SLC22A1 | NR1H2 | ALBU | CBR1 | PADI4 | CP2C8 | HDAC6 |
| PDE6D | DHB11 | PNMT | RET4 | DCK | TGM2 | PYGL |
| CASP7 | CAT | ESR1 | AMPM2 | HYES | GLYC | ELANE |
| THRB | GOT1 | ADH1B | CDD | ERBB4 | IMDH2 | MMP3 |
| KIF11 | MPO | CFAD | CATK | ZAP70 | AKT2 | SLC22A6 |
| CD40 | SLC10A2 | FNTA | ST1E1 | ADK | HEM2 | CAH2 |
| HMOX1 | HDAC2 | MAOM | MET | A1AT | LEG3 |  |
| NOS3 | CPT1B | BMP7 | RENI | CDK6 | NEP |  |
| IMPDH2 | NGAL | RXRA | EPHB4 | HDAC3 |  |  |
| MAPK10 | PLA2G4A | ALOX5 | HDAC3 | MK08 |  |  |
| ALOX5 | HSD11B1 | CMA1 | FKB1A | HCK |  |  |
| HDAC3 | ALDR | MAPK8 | ANDR | IMPDH2 |  |  |
| ADORA2A | TNFRSF1A | ADORA2A | MK10 | EPHB4 |  |  |

**Supplementary Table S2.**

Supplementary Table 2 Differentially expressed genes (DEGs) of atherosclerosis

Differentially expressed genes (DEGs) of atherosclerosis

| CNTN4 | PDE8B | STAB1 | SCFV | FRK | RASGRP3 | S100A8 |
| --- | --- | --- | --- | --- | --- | --- |
| MPP6 | PLD5 | VAV3 | SCUBE3 | IGKC | KYNU | MOP- 1 |
| RAB23 | TC2N | MYOM1 | NAP1L2 | SEMA3D | VWF | TREM1 |
| CAB39L | HSPB7 | CCR1 | IL1RN | TFEC | GPR183 | CHI3L1 |
| IL31RA | PRUNE2 | IGHM | IFI44 | IL2RG | SLAMF7 | ITLN1 |
| MARK1 | FILIP1 | SCFV | IL10RA | IFI30 | SFRP1 | RGS1 |
| CASQ2 | MPP7 | NPNT | CXorf21 | KMO | MS4A4A | CYP1B1 |
| MTUS2 | ANGPTL1 | TTLL7 | LDOC1 | IGKC | SHC4 | ACADL |
| SLC22A3 | PGD | LAIR1 | MCTP1 | IGKC | PLA2G7 | RPS6KA6 |
| LAYN | LGALS9C | SBSPON | ADCY5 | CD52 | FBP1 | PGM5 |
| CATSPERB | RYR2 | IGHM | IGHV3-48 | CD180 | CXCL10 | CNTN3 |
| FHL5 | SEL1L3 | NT5DC3 | CD84 | IGK | LILRB4 | PCDH11Y |
| IBSP | SLC2A12 | IGKC | GULP1 | LY86 | SNX10 | PLCB4 |
| CNN1 | PGR | IGHA1 | CD68 | EMCN | ADGRL4 | LRRN1 |
| CNTN1 | TMEM47 | NPR3 | LOC10272  3407 | AQP9 | MREG | TMEM56-R  WDD3 |
| MYOCD | AKAP6 | CD300A | DDX60L | IGKC | SLC28A3 | PCDH11Y |
| NEGR1 | PARM1 | IGKC | ITGB2 | FABP5P3 | ACP5 | STEAP4 |
| FABP4 | C1orf162 | TM4SF18 | FREM1 | CYTIP | ST14 | TM4SF19 |
| METTL24 | LINC00670 | PLEK | PIK3AP1 | FABP5P3 | LYZ | ADAMDEC1 |
| MX2 | DPP4 | ACKR4 | IGKC | IGKC | CRISPLD1 | MMP12 |
| GPC3 | NPR3 | RNASE1 | IGK | IGKC | C7 |  |
| NEXN | PRUNE2 | IGKC | SCFV | FAM19A2 | MMP7 | KYNU |
| PDZRN3 | NRK | NPL | NPR1 | JCHAIN | ATP6V0D2 | VWF |
| LGALS9C | PGM5P2 | SCFV | ITGAM | C1QTNF7 | CR1 | ADGRL3 |
| TCEAL2 | IGKC | SYNPO2 | PLN | PLIN2 | SULT1C2 | NLRC4 |
| PCDH20 | UNC13C | TDO2 | NPY1R | IGKC | GRIA2 | HEY2 |
| FIBIN | CD163 | DCSTAMP | IGHV3-69-  1 | LPL | LINC00312 | SNORD116-2  1 |
| PDZRN4 | PRDM1 | HAVCR2 | ADGRF5 | MPPED2 | MMP8 | SORBS1 |
| HSPB8 | NCKAP1L | C2 | MMRN1 | GRIA1 | FCER1G | VAMP8 |
| HMOX1 | PGM5P2 | LMOD1 | ATRNL1 | MMP9 | THRB | CCL8 |
| NLGN1 | SCRG1 | IGLJ3 | LGI1 | SCFV | SELE | ACTC1 |
| ANPEP | CD36 | CD4 | AOC3 | MME | C8orf34 | SLA |
| CD4 | AOC3 | MME | C8orf34 | TPH1 | MRAP2 | ITGAX |
| ZNF204P | IGKC | MYEF2 |  |  |  |  |

**Supplementary Table S3**

Supplementary Table 3 Differentially expressed genes (DEGs) of Non small cell lung cancer(NSCLC)

| Differentially expressed genes (DEGs) of Non small cell lung cancer(NSCLC) | | | | | | |
| --- | --- | --- | --- | --- | --- | --- |
|  |  |  |  |  |  |  |
| GPM6A | PLAC9 | FMO2 | CD93 | OLR1 | AGTR2 | METTL7A |
| CA4 | AGER | SPOCK2 | SLC39A8 | CRTAC1 | SYNPO | NTN4 |
| ADAMTS8 | SH2D3C | CAVIN2 | GRASP | LMOD1 | ADAMTSL4 | MYH10 |
| RTKN2 | STX11 | ARHGAP31 | IL3RA | C1orf162 | TOP2A | HLA-E |
| FAM107A | TNNC1 | CLEC14A | CSRNP1 | MFAP4 | CCDC69 | IL33 |
| MYZAP | FCN3 | HBA2 | CD34 | MGAT3 | DPYSL2 | ABCA3 |
| GPD1 | HIGD1B | TIE1 | TNS1 | MSR1 | MT1M | FOSB |
| SLC6A4 | MYCT1 | C14orf132 | DLC1 | HYAL1 | CD52 | NCF2 |
| TEK | ADRB2 | CYYR1 | PLA2G4F | LAMP3 | VSIG4 | TK1 |
| GPIHBP1 | WWC2 | FAM162B | ESAM | PID1 | ARHGEF26 | SELPLG |
| ITLN2 | NPR1 | SLIT3 | KANK2 | CFD | ALOX5 | GMFG |
| TCF21 | S1PR1 | SPN | ACE | GPX3 | DUOX1 | HK3 |
| KANK3 | RAMP2 | RAI2 | CHRDL1 | DOK2 | SUSD2 | HSPB8 |
| EDNRB | INMT | HSPB6 | TRPV2 | TNS2 | KLF6 | ACP5 |
| LIMS2 | PTPRB | CALCRL | CAT | MYRF | UBE2C | MYBL2 |
| ROBO4 | FAM189A2 | CCL23 | WFS1 | PPP1R14A | GNG11 | NDNF |
| JAM2 | FOXF1 | ITGA8 | FGR | LPL | CYBRD1 | CDCA8 |
| VEGFD | CDH5 | AOC3 | GKN2 | SLC46A2 | CAV2 | GAS6 |
| CLIC5 | FGD5 | CD36 | SLC1A1 | ANKRD29 | A2M | LMO7 |
| FHL5 | CLDN18 | MMRN2 | OSCAR | C5AR1 | TACC1 | NEDD9 |
| PRX | GIMAP8 | SLIT2 | VWF | HEG1 | WIF1 | NUSAP1 |
| LDB2 | LGI3 | FGFR4 | HBB | IL1RL1 | CDC20 | CSF3 |
| HSPA12B | RAMP3 | CGNL1 | AQP4 | MRC1 | CCNB1 | CYP27A1 |
| ANKRD1 | VIPR1 | EPAS1 | KCNK3 | CYP4B1 | PPP1R14B | GIMAP4 |
| GDF10 | TNXB | TBX4 | RASIP1 | PDK4 | KLF9 | MCM4 |
| CLEC3B | BTNL9 | HHIP | CACNA2D2 | CD300LF | LTBP4 | CTHRC1 |
| MCEMP1 | ADH1B | LRRC32 | SFTPC | GIMAP7 | TPPP3 | MMP19 |
| PTPN21 | VEPH1 | SELP | TMEM204 | UBE2T | ANXA3 | C7 |
| ACVRL1 | FABP4 | JCAD | GYPC | MS4A15 | MYADM | SPARCL1 |
| FHL5 | RGCC | OTUD1 | MS4A7 | PYCR1 | ALOX5AP | ADGRE5 |
| FHL1 | ABI3BP | CLDN5 | GATA6 | ACKR1 | RBP4 | GADD45B |
| ARHGE15 | SCN7A | COX4I2 | COX7A1 | TENT5B | ZWINT | SPI1 |
| SEMA3G | ADRB1 | WISP2 | HSD17B6 | VSIR | CAVIN1 | RRM2 |
| TMEM100 | DES | CAV1 | PEBP4 | TGFBR2 | CD83 | CLIC3 |
| EFCC1 | MAMDC2 | SPTBN1 | LHFPL6 | KLF2 | PPP1R15A | HMGA1 |
| GRK5 | EMP2 | GIMAP6 | PLA2G1B | COLEC12 | NME1 | BIRC5 |
| ECSCR | UPK3B | ANOS1 | FBLN5 | ITM2A | TPX2 | SEMA3B |
| LYVE1 | PECAM1 | GLIPR2 | OGN | LRRK2 | LIMCH1 | ATOH8 |
| MYL9 | SFTPD | DEPP1 | CYBB | TMEM125 | NECTIN1 | SOX2 |
| PAFAH1B3 | CCNA2 | ADGRF5 | MMP28 | RNASE1 | SLC6A8 | AKR1B10 |
| GJA5 | NPNT | SOD3 | CTGF | GJB2 | SLC34A2 | PKP1 |
| C11orf96 | ADAMTS1 | SCGB1A1 | LRRN4 | HLA-DPA1 | SFTPB | GJB6 |
| KPNA2 | JUND | MCM2 | CAMK2N1 | THBS2 | SCGB3A2 | PTHLH |
| MFSD2A | ID4 | SCEL | MGP | FOXA2 | NAPSA | SPRR1B |
| MAOB | CDCA5 | TYROBP | THBD | SRGN | PIGR | TP63 |
| KIF2C | KIFC1 | MDK | HIST1H2BD | HLA-DPB1 | MT-ND6 | DSG3 |
| ANLN | RAB11FIP1 | ATP13A4 | DPT | APOC1 | SERPINA1 | CALML3 |
| KCTD12 | SPP1 | ITLN1 | HLA-DOA | FAM83A | TNS4 | NKX2- 1 |
| ARRB1 | TPSAB1 | SFTPA2 | RPL39L | MMP1 | PFN2 | KRT6B |
| IL7R | OTULINL | TRIP13 | ALDH3B1 | TMPRSS2 | GJB3 | TRIM29 |
| FIBIN | ALDH2 | SELENOP | MMP11 | B3GNT3 | LYPD3 | EEF1A2 |
| FPR1 | PMP22 | SELENBP1 | DCN | LYZ | ITGB4 | DSC3 |
| CEP55 | EDN1 | S100A4 | RHOBTB2 | COL3A1 | AGR3 | AKR1C2 |
| IQANK1 | PRC1 | DRAM1 | CTSH | SCNN1B | HLA-DQB1 | CES1 |
| SLCO2B1 | CDK1 | LAD1 | FAM83D | HLA-DRB5 | GPX2 | CLDN1 |
| CCNB2 | RRAS | CYR61 | FOS | ALOX15B | S100A2 | CXCL17 |
| PLK1 | AURKB | CRABP2 | LAPTM5 | HLA-DRA | HOPX | LY6D |
| SLCO2A1 | TSPAN12 | TMPRSS4 | FCGR3A | HLA-DRB1 | SFTA2 | MMP10 |
| AQP1 | HMGB3 | TPSB2 | PGC | C1orf116 | PRAME | KRT5 |
| PAPSS2 | CXCL2 | C1QA | RRAD | STEAP4 | COL17A1 | SPRR1A |
| MNDA | DUSP1 | GPRC5A | DMBT1 | ICAM1 | SFN | SPRR2D |
| GGTLC1 | EFEMP1 | DSP | IL6 | EIF4EBP1 | MMP9 | CLCA2 |
| CHI3L2 | CPA3 | CITED2 | CDH3 | C1QC | SERPINB5 | KRT14 |
| LIFR | SLC2A1 | SLC9A3R2 | PODXL2 | CD55 | CA9 | CEACAM5 |
| PTGDS | NPM3 | ETV4 | COL1A1 | SCGB3A1 | CST1 | MSLN |
| LY86 | SFTPA1 | CYB5A | MMP12 | PERP | COL7A1 | SPRR2A |
| VSIG2 | CXCL16 | ALPL | COL10A1 | PITX1 | C15orf48 | SPRR3 |
| LDLR | FOXM1 | SLC7A5 | CA2 | PLAU | GPC3 | S100P |
| NES | NECTIN4 | MYH11 | PROM2 | TSPAN7 | KRT6A | S100A7 |
| PRELP | FBP1 | FERMT1 | FOLR1 | SFTA3 | CXCL13 | PI3 |
| CADM1 | ECT2 | C1QB | CD163 | C16orf89 | CXCL14 | KRT13 |
| CBLC | AHNAK | EHD2 | TGM2 | GPR87 | KRT15 | FLRT3 |
| PROS1 | PSAT1 | KLF4 | CTSS | PTGES | C3 | PRG4 |
| HBEGF | GAPDH | LARGE2 | TXNIP | CD74 | UCHL1 | ENG |
| SERPING1 | NR4A1 | FCER1G | RHOV | CPM | NQO1 | MARCO |
| PTTG1 | NRGN | C2 | TREM1 | HPGD | KRT17 | CPB2 |
| ASF1B | ZFP36 | C20orf85 | C4BPA | SLPI | KRT16 | RETN |
| BMP2 | VIM | ALG1L | EGR1 | IGFBP3 | GJB5 | EMCN |
