# Supplementary material for: Prediction and validation of common targets in atherosclerosis and non-small cell lung cancer influenced by atorvastatin
Source: BMC Complement Med Ther. 2023 Nov 17;23:415. doi: 10.1186/s12906-023-04255-7 (PMC10657002; doi:10.1186/s12906-023-04255-7)
Supplement: Supplementary file 2 — Additional file 2. [file 12906_2023_4255_MOESM2_ESM.docx]

Supplementary Materials for

**Prediction and validation of common targets in atherosclerosis and non-small cell lung cancer influenced by atorvastatin**

**Yuqian Li^1^, Luyao Li^1^, Xue Yang^1^, Qiqi Lei^1^, Liuyan Xiang^1^, Yuanru Wang^1^, Simeng Gu^2^, Yajun Cao^1^, Lu Tie^2^, Yan Pan^2^****, Xuejun Li^1, 2*^**

^1^Department of Pharmacology, School of Pharmacy, Shihezi University, Shihezi 832002, China

^2^Department of Pharmacology, School of Basic Medical Sciences, Peking University, Beijing 100191, China

*Correspondence: Xuejun Li, Email: xjli@bjmu.edu.cn.

^1^Department of Pharmacology, School of Pharmacy, Shihezi University, Shihezi 832002, China

^2^Department of Pharmacology, School of Basic Medical Sciences, Peking University, Beijing 100191, China

Supplementary Original western blot images for Figure 6D. Original western blotting for MMP9, MMP12, CD36 and FABP4 of the atorvastatin treated A549 cells. The protein blots are imaged by X-ray film exposure.

Figure 6D


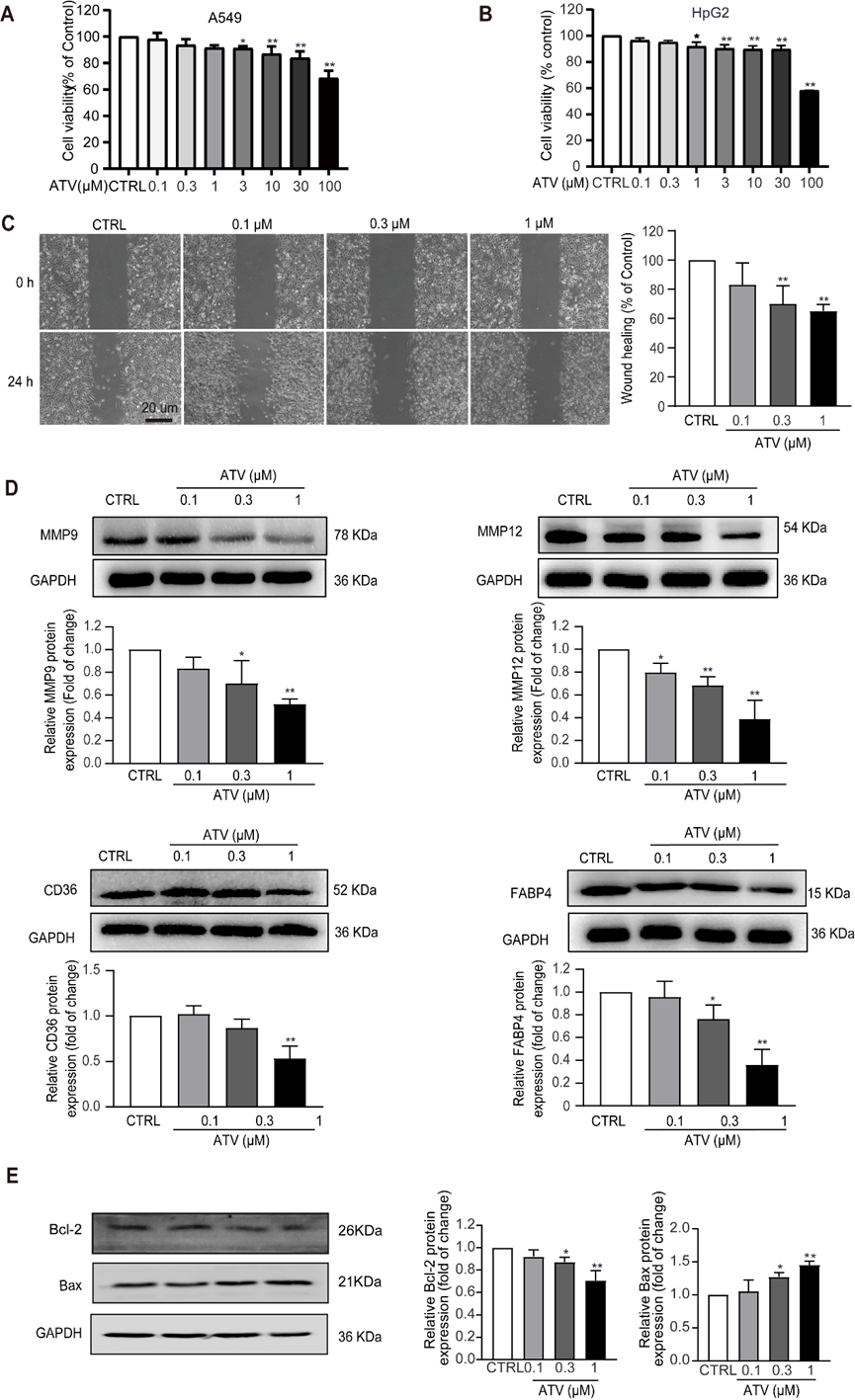


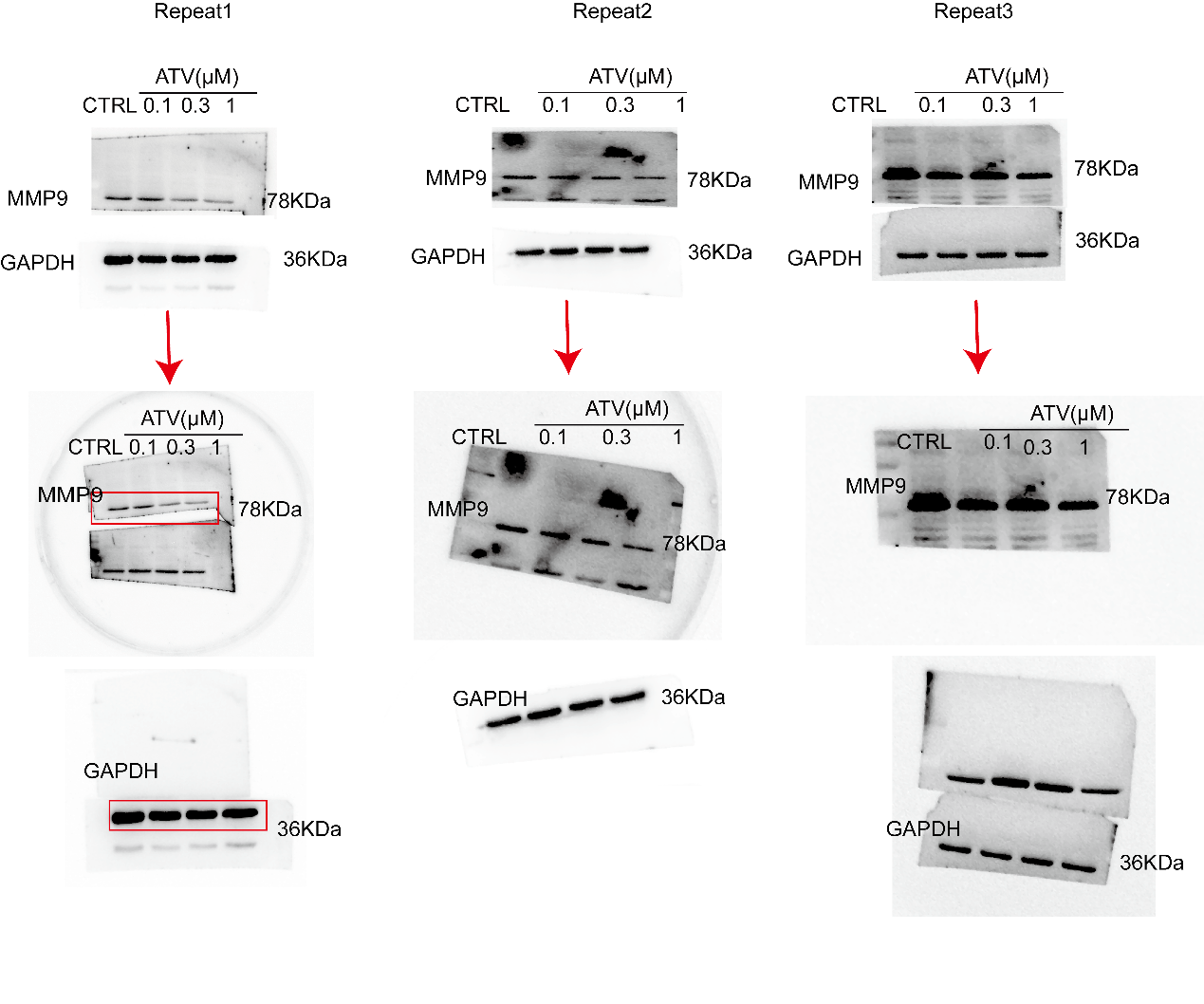


Fig. 6D: ATV-MMP9


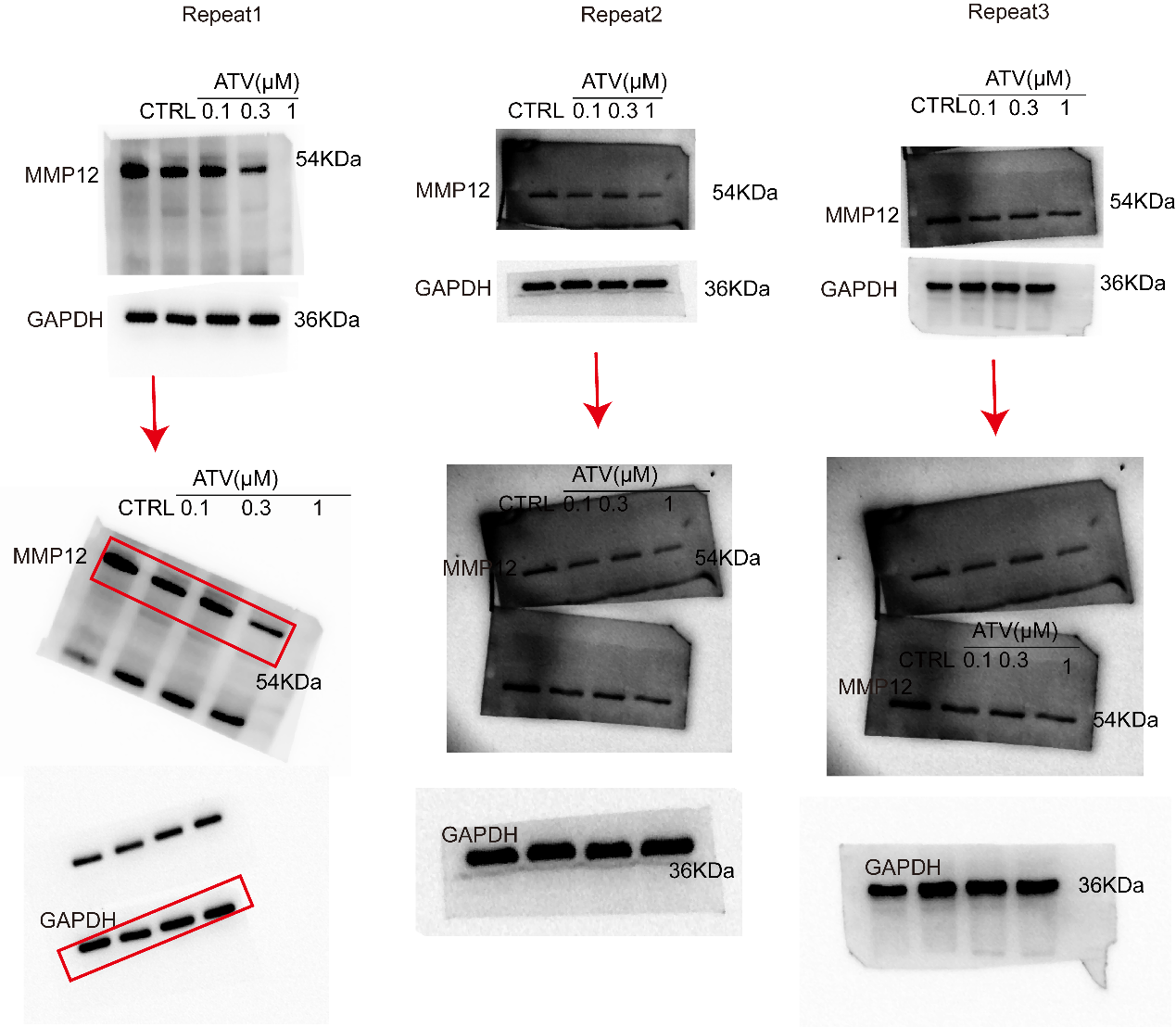


Fig. 6D: ATV-MMP12


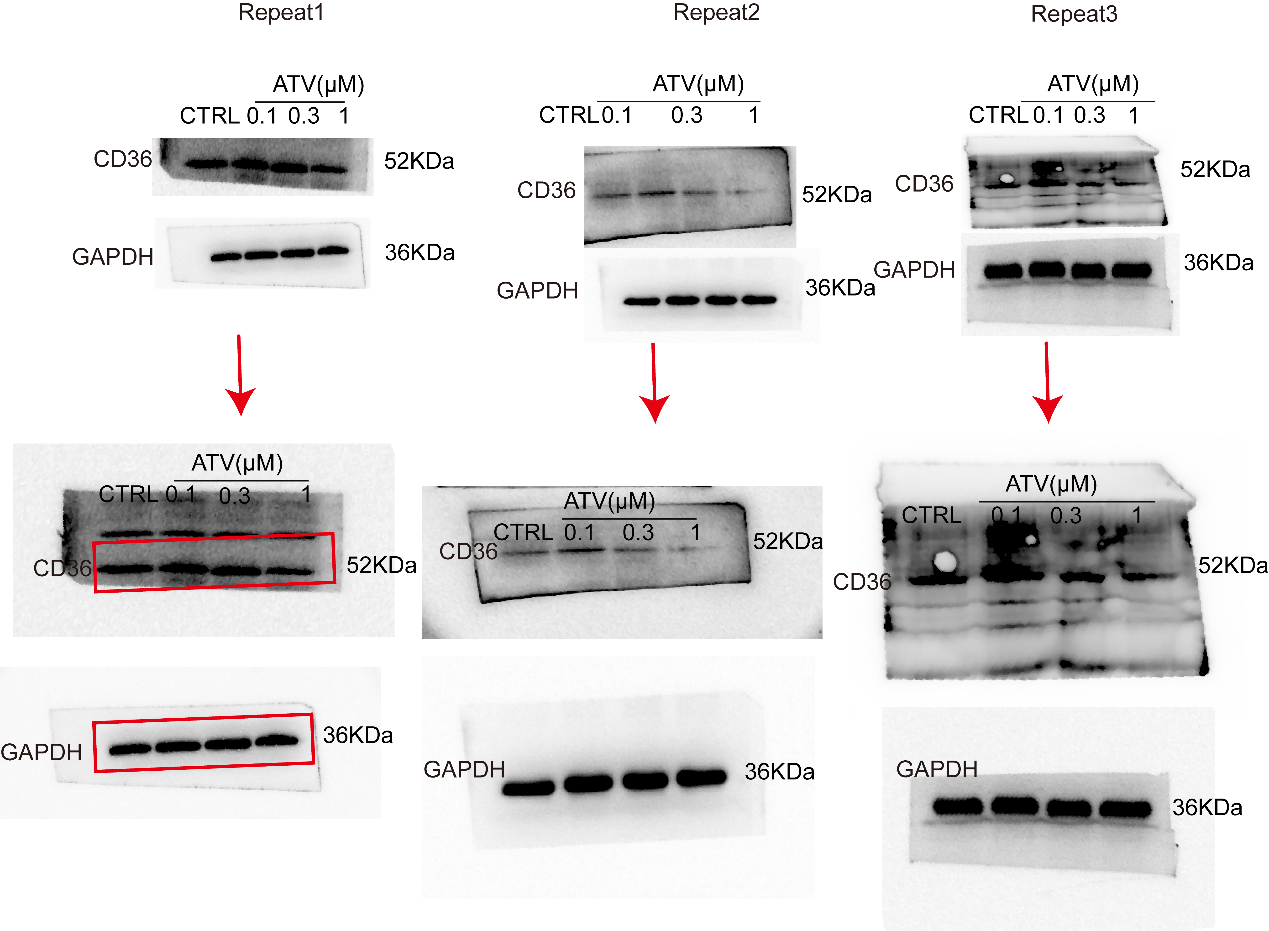


Fig. 6D: ATV-CD36


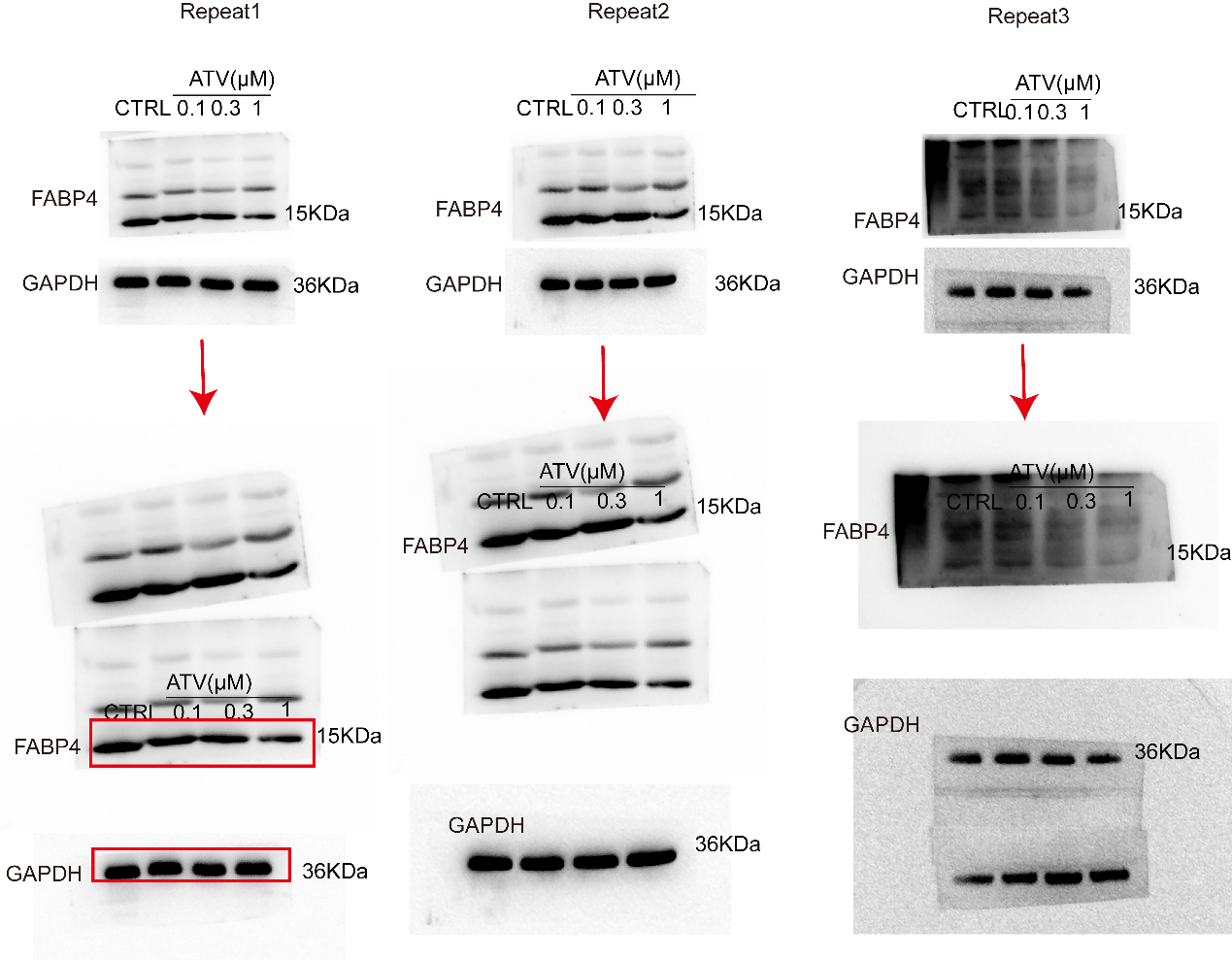


Fig. 6D: ATV-FABP4

Supplementary Original western blot images for Figure 6E. Original western blotting for Bcl-2 and Bax of the atorvastatin treated A549 cells. The protein blots are imaged by X-ray film exposure.

Figure 6E


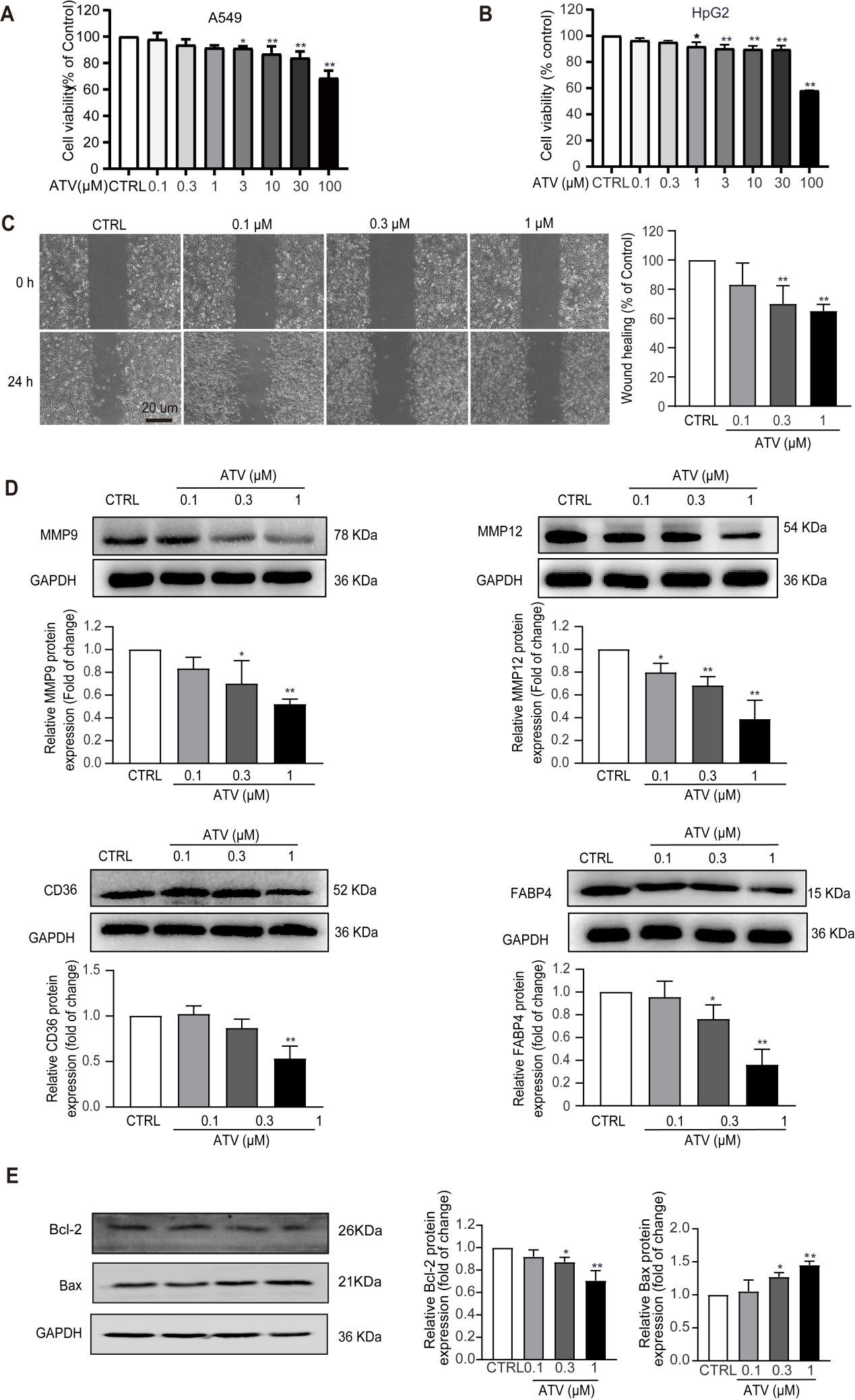


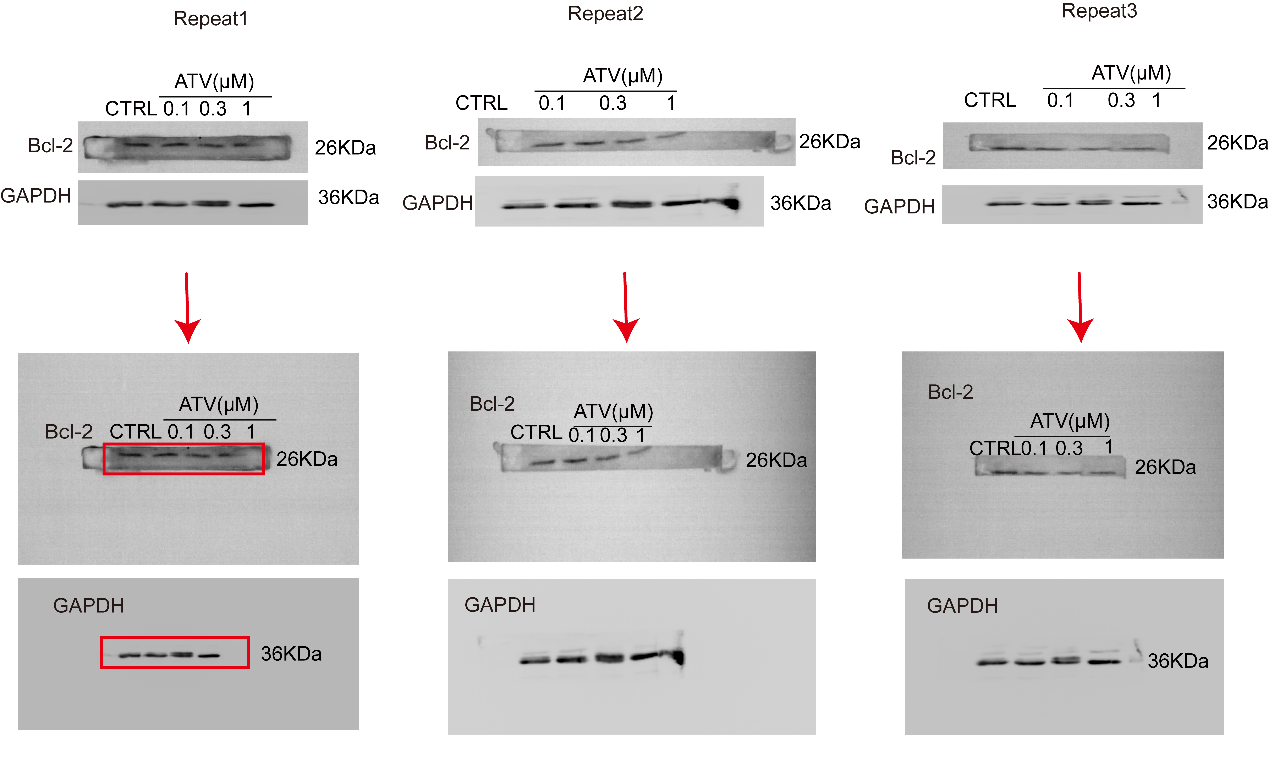


Fig. 6E: ATV-Bcl-2


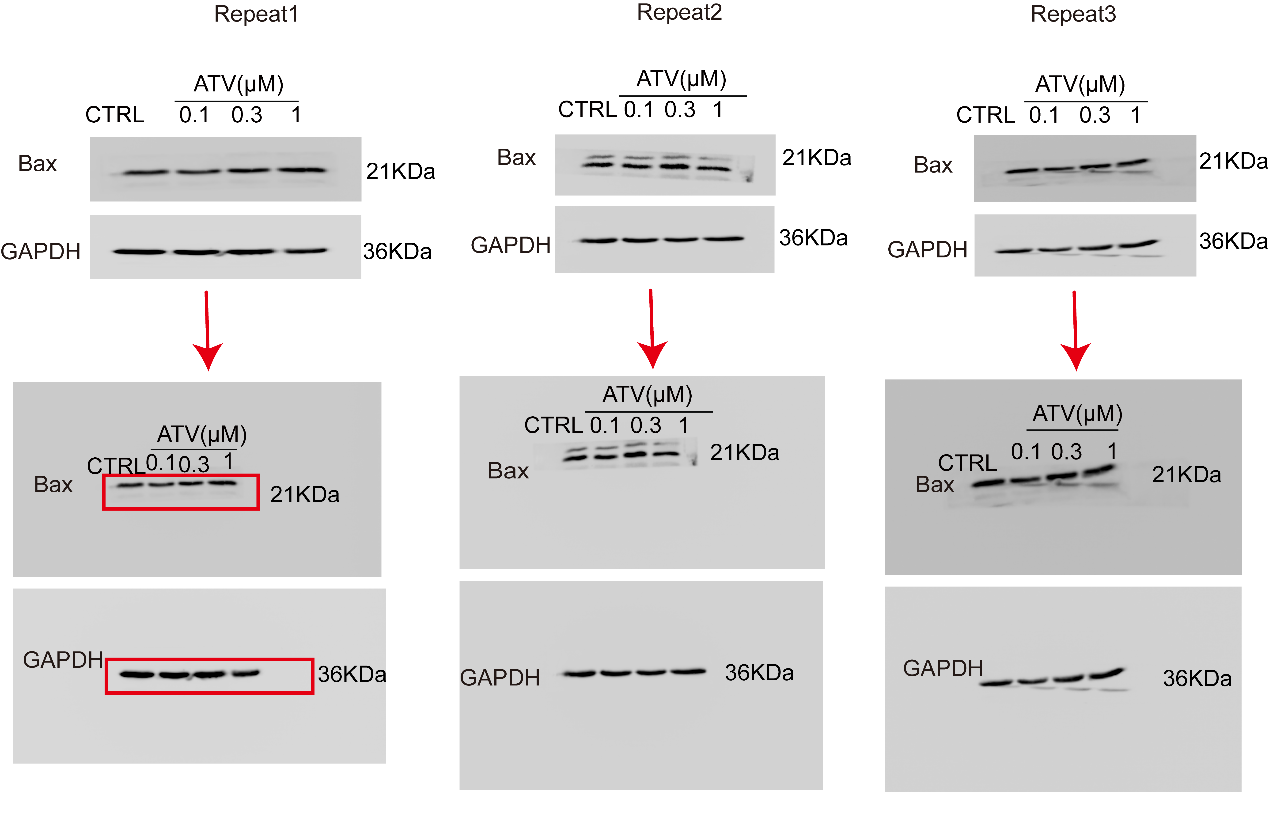


Fig. 6E: ATV-Bax
