# Supplementary material for: Prediction and validation of common targets in atherosclerosis and non-small cell lung cancer influenced by atorvastatin
Source: BMC Complement Med Ther. 2023 Nov 17;23:415. doi: 10.1186/s12906-023-04255-7 (PMC10657002; doi:10.1186/s12906-023-04255-7)
Supplement: Supplementary file 3 — Additional file 3. [file 12906_2023_4255_MOESM3_ESM.docx]

Supplementary Materials for

**Prediction and validation of common targets in atherosclerosis and non-small cell lung cancer influenced by atorvastatin**

**Yuqian Li^1^, Luyao Li^1^, Xue Yang^1^, Qiqi Lei^1^, Liuyan Xiang^1^, Yuanru Wang^1^, Simeng Gu^2^, Yajun Cao^1^, Lu Tie^2^, Yan Pan^2^****, Xuejun Li^1, 2*^**

^1^Department of Pharmacology, School of Pharmacy, Shihezi University, Shihezi 832002, China

^2^Department of Pharmacology, School of Basic Medical Sciences, Peking University, Beijing 100191, China

*Correspondence: Xuejun Li, Email: xjli@bjmu.edu.cn.

^1^Department of Pharmacology, School of Pharmacy, Shihezi University, Shihezi 832002, China

^2^Department of Pharmacology, School of Basic Medical Sciences, Peking University, Beijing 100191, China

Supplementary Original western blot images for Figure 2D. Original Oncomine database for the expression of four common targets MMP9, MMP12, CD36 and FABP4 in various cancers.

Figure 2D


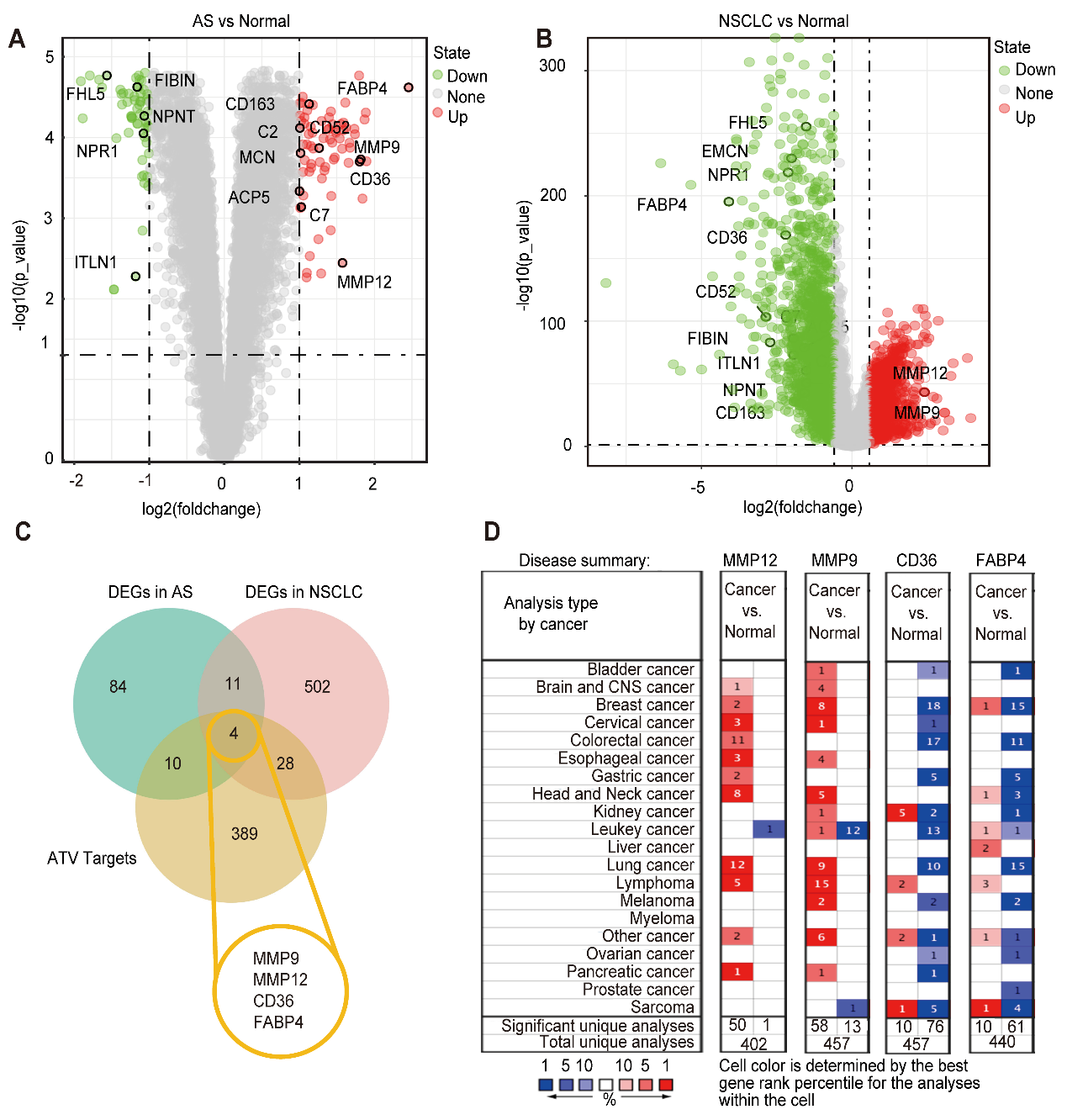


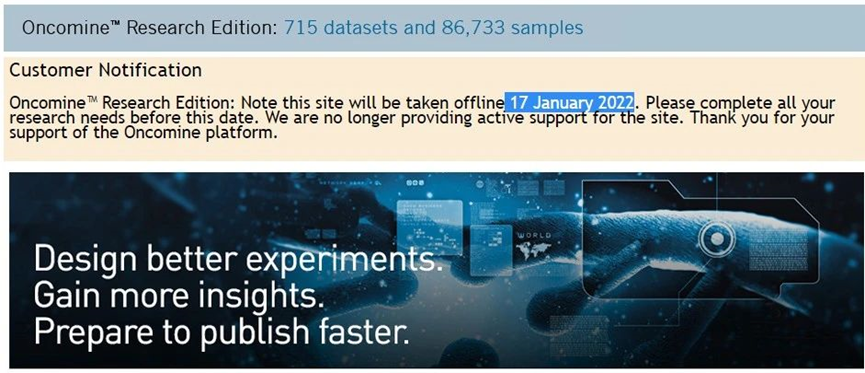


Oncomine TM Research Edition(https://www.oncomine.org/resource/main.html): 715 datasets and 86,733 samplesCustomer Notification.

Oncomine TMResearch Edition: Note this site will be taken offline 17 January 2022.


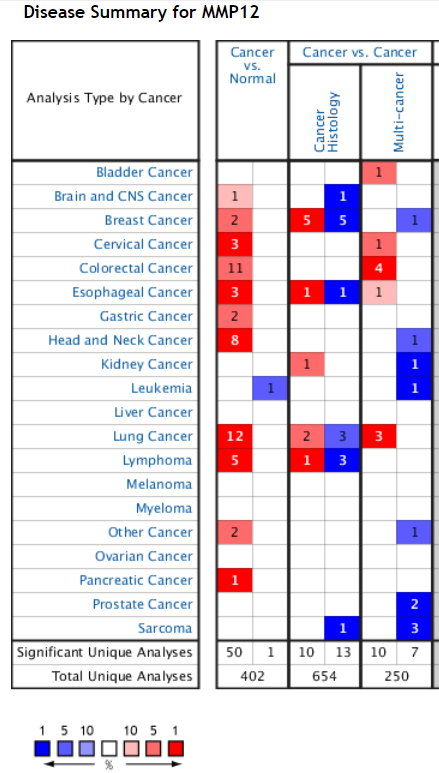


Fig. 2D: Oncomine database - MMP12


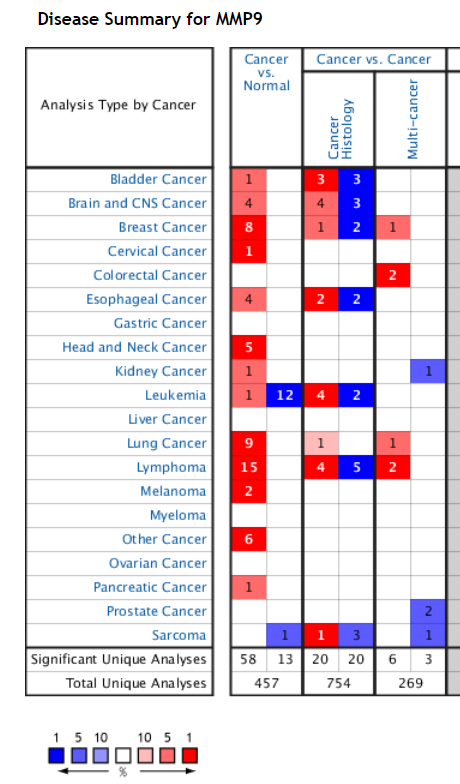


Fig. 2D: Oncomine database -MMP9


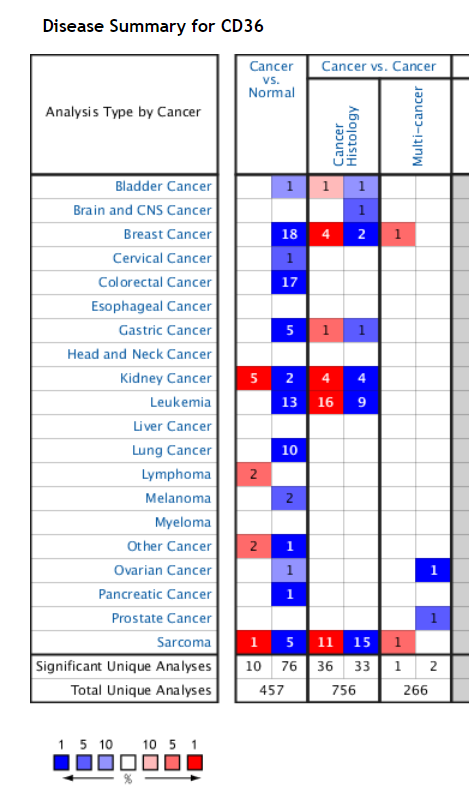


Fig. 2D: Oncomine database -CD36


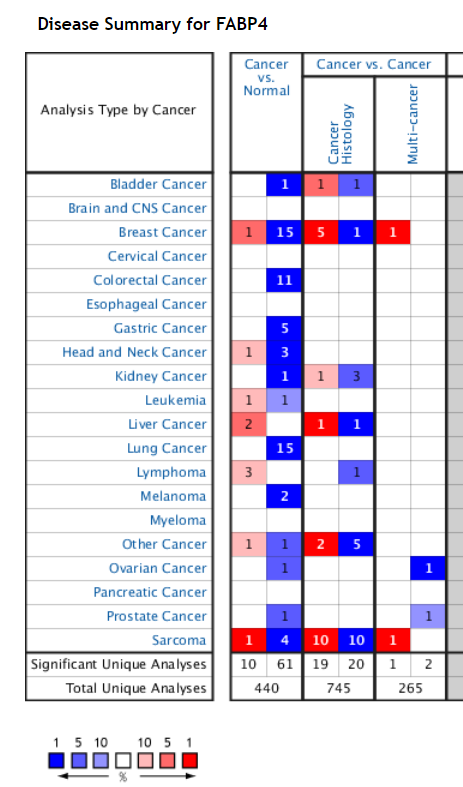


Fig. 2D: Oncomine database -FABP4
